# EVA1A regulates hematopoietic stem cell regeneration via ER-mitochondria mediated apoptosis

**DOI:** 10.1038/s41419-023-05559-9

**Published:** 2023-01-30

**Authors:** Bo Liu, Yuanyuan Zhou, Qiaofeng Wu, Yuting Fu, Xianli Zhang, Zhenkun Wang, Weiwei Yi, Hu Wang, Zhiyang Chen, Zhangfa Song, Wei Xiong, Yugang Qiu, Weifeng He, Zhenyu Ju

**Affiliations:** 1 Key Laboratory of Regenerative Medicine of Ministry of Education, Institute of Aging and Regenerative Medicine, Jinan University, Guangzhou, 510632 China; 2 Institute of Aging Research, Hangzhou Normal University School of Medicine, Hangzhou, 310036 China; 3 Department of Colorectal Surgery, Sir Run Run Shaw Hospital, Zhejiang University, Hangzhou, 310016 China; 4 Hefei National Laboratory for Physical Sciences at the Microscale, Institute on Aging and Brain Disorders, Division of Life Sciences and Medicine, University of Science and Technology of China, Hefei, 230026 China; 5 School of Rehabilitation Medicine, Weifang Medical University, Weifang, 261053 China; 6 Institute of Burn Research, Southwest Hospital, State Key Laboratory of Trauma, Burn and Combined Injury, Chongqing Key Laboratory for Disease Proteomics, Army Military Medical University, Chongqing, 400038 China

**Keywords:** Regeneration, Apoptosis, Haematopoietic stem cells, Endoplasmic reticulum, Mitochondria

## Abstract

Excessive protein synthesis upon enhanced cell proliferation frequently results in an increase of unfolded or misfolded proteins. During hematopoietic regeneration, to replenish the hematopoietic system, hematopoietic stem cells (HSCs) are activated and undergo a rapid proliferation. But how the activated HSCs respond to the proliferation pressure is still ambiguous; The proper control of the functional reservoir in the activated HSCs remains poorly understood. Here, we show a significant upregulation of EVA1A protein associated with the increase of ER stress during hematopoietic regeneration. Deletion of *Eva1a* significantly enhances the regeneration capacity of HSCs by inhibiting the ER stress-induced apoptosis. Mechanistically, the expression of EVA1A protein was upregulated by CHOP, and thereby promoted the ER-mitochondria interlinking via MCL1, which resulted in mitochondria-mediated apoptosis. These findings reveal a pathway for ER stress responses of HSCs by the EVA1A mediated apoptosis, which play an important role in HSCs regeneration.

## Introduction

The hematopoietic system is sustained by hematopoietic stem cells (HSCs), which have the capacity for self-renewal and differentiating into all lineages of mature blood cells. Although HSCs are mostly quiescent during homeostasis, they can be quickly activated to replenish the hematopoietic system in response to various stresses, such as blood loss, infections/inflammation or chemotherapy-induced myelotoxicity [1, 2]. The regeneration capacity of HSCs is affected by multiple stress stimuli, including reactive oxygen species (ROS), nutrient fluctuation, DNA damage, and ER stress [3–5]. HSCs maintenance during homeostatic state has been widely investigated, however the proper control of the functional reservoir in the activated HSCs remains poorly understood.

The endoplasmic reticulum (ER) is an organelle responsible for many cellular processes, such as protein folding and secretion, lipid synthesis, and calcium storage. ER membrane physically and functionally interacts with many intracellular membranous structures that put ER at a great position to sense cellular perturbations and activate signaling pathways to restore homeostasis [6, 7]. Many sources of stress, such as viral infections, ROS, and proliferative signals, cause misfolded proteins accumulation in ER and subsequently activate unfolded protein response (UPR) to restore the protein-folding homeostasis by reducing protein synthesis and increasing protein-folding and degradative capacities of ER [8, 9]. But if the UPR cannot resolve the stress, apoptosis will be initiated to clear the damaged cells.

Although HSCs have profoundly restricted protein synthesis, recent studies suggest an essential role of ER stress response/UPR in HSCs regulation [10–12]. Activation of the ER stress signaling IRE1α–XBP1 preserves HSC self-renewal after exposure to LPS [10]. Reducing ER stress levels by tauroursodeoxycholic acid maintains functional HSCs in vitro [11]. Alleviation of ER stress by bile acids is required for HSC expansion during fetal hematopoiesis [12]. In addition, HSCs are more susceptible to ER stress and initiate apoptosis, which may contribute to the HSC pool clonal integrity by selectively removing damaged HSCs [5, 11]. To date, despite the significance of ER stress response in HSCs biology is approved, how ER stress regulates HSCs apoptosis and the molecular mechanism remains to be elucidated.

EVA1A, also known as TMEM166 (transmembrane protein 166) or FAM176A (family with sequence similarity 176 member A), is an ER membrane protein involved in autophagy and apoptosis [13]. It has been shown that EVA1A is elevated during embryonic neurogenesis and enhances neural stem cells self-renewal via promoting autophagy [14]. However, the connection between EVA1A and apoptosis in stem cell biology is still unknown. In this study, our data show a significant increase of ER stress with an enhanced expression of EVA1A protein in hematopoietic stem/progenitor cells (HSPCs) under hematopoietic pressure. Deletion of *Eva1a* significantly enhances the self-renewal and repopulation capacity of HSPCs in mice via inhibiting the ER stress-induced apoptosis. The mechanism investigation shows during ER stress, C/EBP homologous protein (CHOP) upregulated the expression of EVA1A protein, which promoted the ER-mitochondria-mediated apoptosis by interacting with MCL1.

## Results

### ER stress promotes EVA1A expression via CHOP

**Fig. 1:**
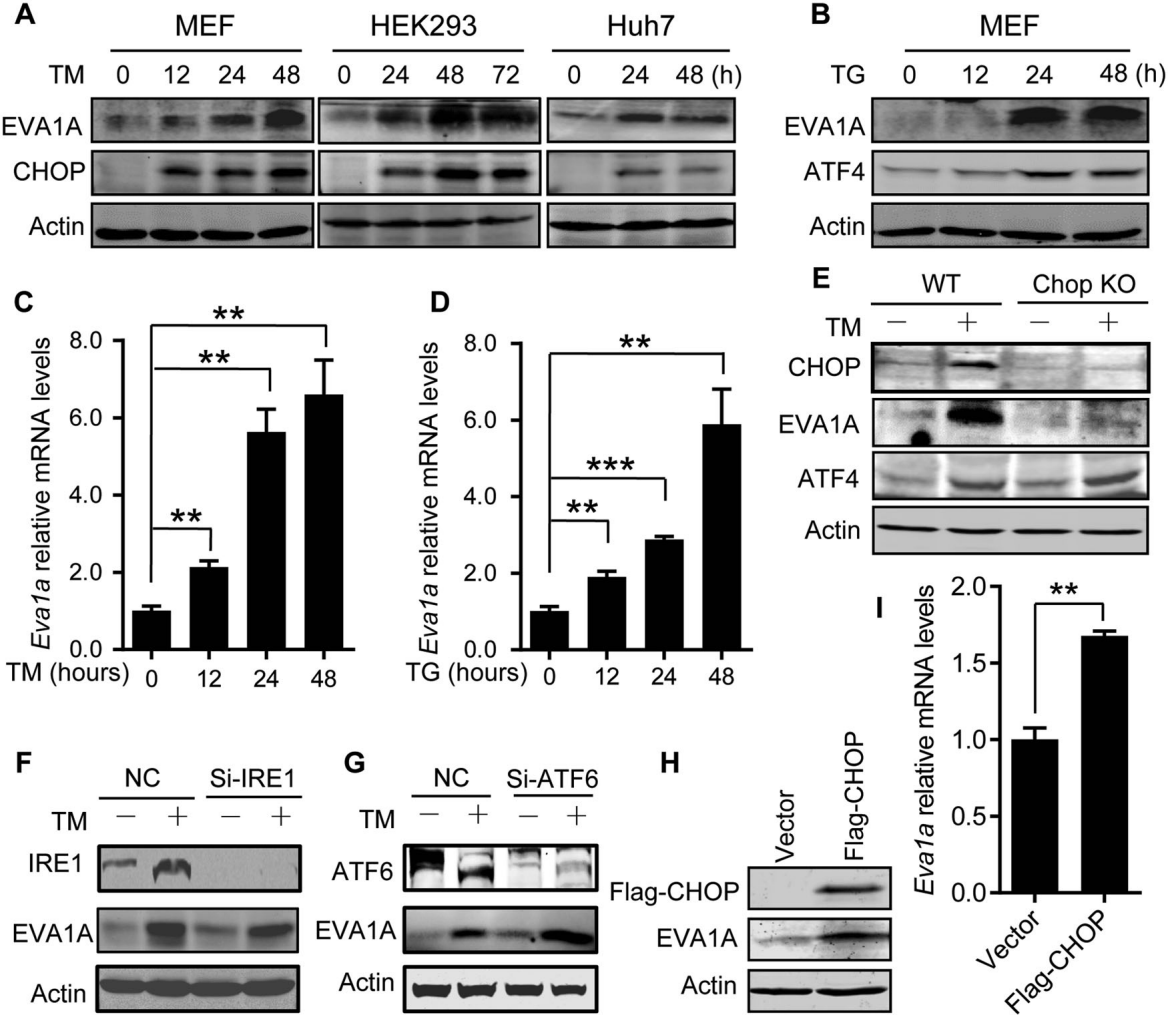
ER stress promotes EVA1A expression via CHOP. **A** Western blot analysis of CHOP and EVA1A protein levels at indicated time points in cells treated with 3 μg/ml Tunicamycin (TM). **B** Western blot analysis of ATF4 and EVA1A protein levels at indicated time points in MEF cells treated with 0.2 μM Thapsigargin (TG). **C**, **D** Relative mRNA level of EVA1A in MEF cells treated with TM (**C**) or TG (**D**) at indicated time points (*n* = 3). **E** The protein level of EVA1A in WT and *Chop* KO MEF cells treated without or with 3 μg/ml TM for 36 h. **F, G** The protein level of EVA1A in IRE1 knockdown (**F**) or ATF6 knockdown (**G**) MEF cells treated without or with 3 μg/ml TM for 36 h. **H** Western blot analysis of the EVA1A protein level in Flag-CHOP overexpressing HEK293 cells. **I** The relative mRNA levels of EVA1A in HEK293 cells overexpressing Flag-CHOP (*n* = 3). Data are presented as mean ± SD, (**p < 0.01, and ***p < 0.001).

EVA1A is known as an ER membrane protein, but little is known about its role in the function of ER. To investigate a potential role of EVA1A in ER, we measured the change of EVA1A expression during ER stress. Interestingly, with the ER stress response, increased protein levels and mRNA levels of EVA1A were detected in cells treated with two chemical inducers of ER stress: thapsigargin and tunicamycin (Fig. 1A-D), suggesting a role of EVA1A in ER stress response.

EVA1A is an ER membrane protein involved in apoptosis [13]. CHOP is the major transcription factor regulating ER stress-induced apoptosis. To investigate a potential role of CHOP in the ER stress-induced EVA1A expression, the expression of EVA1A were assessed in *Chop* knockout cells treated with or without tunicamycin. The results showed a significant increase of EVA1A protein level in the wild type (WT) cells, but not in *Chop* knockout cells upon tunicamycin treatment (Fig. 1E). Interestingly, the upregulation of EVA1A protein induced by tunicamycin treatment was not affected by IRE1 and ATF6 knockdown (Fig. 1F, G). Consistently, overexpression of CHOP dramatically promoted the expression of EVA1A (Fig. 1H, I). Taken together, these data indicate that ER stress promotes EVA1A expression via CHOP.

### ER stress upregulates EVA1A expression during hematopoietic regeneration

**Fig. 2:**
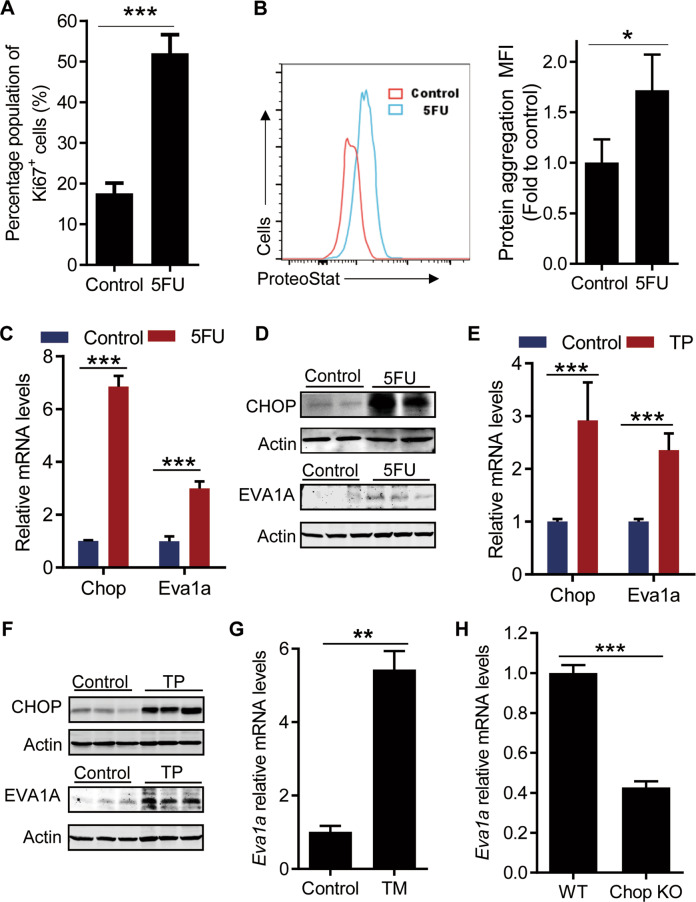
ER stress promotes EVA1A expression during hematopoietic regeneration. **A–D** WT mice were treated with a single dose of 5FU (150 mg/kg) and sacrificed at day 8 posttreatment. **A** The results present the percentage of Ki67-positive cells in SLAM-HSCs (Lin^−^Sca1^+^cKit^+^CD48^−^ CD150^+^) after 5FU treatment (*n* = 4). **B** Representative FACS histogram (left) and quantification (right) of the level of protein aggregation in SLAM-HSCs after 5FU treatment (*n* = 4). **C** Relative mRNA levels of CHOP and EVA1A in LSK (Lin^−^cKit^+^Sca1^+^) cells from WT mice after 5FU treatment (*n* = 9). **D** Western blot analysis of CHOP and EVA1A protein levels in Lin^−^ (Lineage^−^) cells from WT mice after 5FU treatment. **E** Relative mRNA levels of CHOP and EVA1A in LSK cells from lethally irradiated mice after transplanted (TP) with 2 × 10^6^ BM cells 14 days. (*n* = 9) **F** Western blot analysis of CHOP and EVA1A protein levels in Lin^−^ cells from mice as in (**E**). **G** Relative mRNA level of EVA1A in LSK cells from WT mice treated with 0.5 mg/kg Tunicamycin for 1 Week (*n* = 3). **H** The relative mRNA level of EVA1A in LSK cells from WT and *Chop* KO mice (*n* = 3). Data are presented as mean ± SD, (*p < 0.05, **p < 0.01, and ***p < 0.001).

Enhanced cell proliferation frequently results in an accumulation of unfolded and misfolded proteins, which is a major inducing factor of the ER stress [9, 11, 15]. To investigate how the activated HSCs responds to the proliferative stress during hematopoietic regeneration, we checked the ER stress level in HSPCs under hematopoietic stress such as 5FU or transplantation treatment. Consistently with the previous study, we found that 5FU strongly induced SLAM-HSC (Lin^−^cKit^+^Sca1^+^CD48^−^CD150^+^) proliferation (Fig. 2A). Interestingly, with the increase of HSCs proliferation, a significant accumulation of protein aggregation was detected in SLAM-HSCs with 5FU treatment (Fig. 2B), as demonstrated by the enhanced ProteoStat staining, a specific fluorescent dye sensitive for detecting protein aggregation. Meanwhile, a significant increase of the mRNA and protein levels of CHOP, a specific ER stress response protein, were detected in LSK (Lin^−^cKit^+^Sca1^+^) and Lin^−^ (Lineage^−^) bone marrow (BM) cells with 5FU treatment (Fig. 2C, D), suggesting an enhanced ER stress in HSPCs under hematopoietic stress. In addition, with the ER stress response, increased mRNA levels and protein levels of EVA1A were detected in the same HSPCs with 5FU treatment (Fig. 2C, D), suggesting an ER stress-induced EVA1A expression during hematopoietic regeneration.

To further confirm the ER stress-induced EVA1A expression during hematopoietic regeneration, the ER stress and EVA1A expression levels were also assessed in HSPCs with transplantation treatment. The results showed, with the ER stress response, the expression of EVA1A was also dramatically increased in the HSPCs after transplantation (Fig. 2E, F), as demonstrated by the upregulated mRNA and protein levels of EVA1A and CHOP in LSK and Lin^−^ BM cells from lethally irradiated mice transplanted with 2 × 10^6^ BM cells 14 days, consistently with our previous study showing increased proliferation and protein aggregation in the HSPCs after transplantation [16].

Lastly, to further confirm the enhanced EVA1A expression in HSPCs was stimulated by ER stress, the EVA1A expression was further analyzed in LSK cells treated with tunicamycin. The data show an enhanced EVA1A mRNA level in the LSK cells from the mice treated with tunicamycin (Fig. 2G). Consistently, *Chop* knockout also significantly inhibited the expression of EVA1A in LSK cells from *Chop* deficient mice (Fig. 2H). Taken together, these data suggest a role of EVA1A in ER stress response reactions of HSPCs during hematopoietic regeneration.

### *Eva1a* deficiency increases the number of HSPCs

**Fig. 3:**
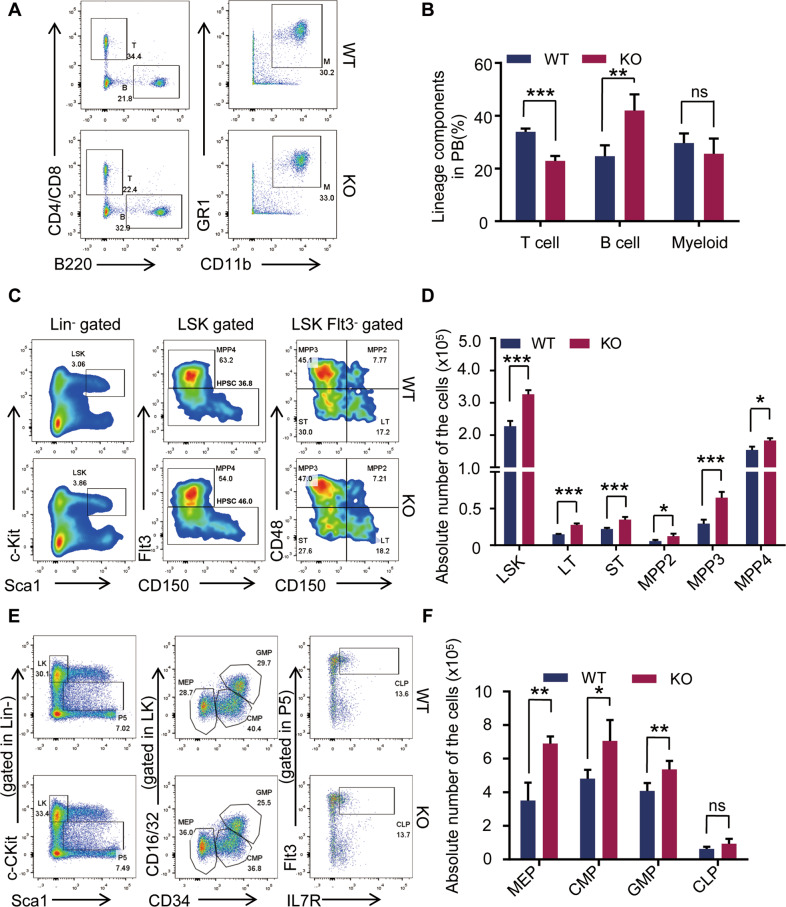
*Eva1a* deficiency increases the number of HSPCs. **A, B** Representative FACS plots (**A**) and Quantification of the composition (**B**) of T (CD4^+^/CD8^+^) cells, B (B220^+^) cells, and myeloid (GR1^+^CD11b^+^) in PB from WT and *Eva1a*^*F*/*F,Vav-Cre*^ mice (*n* = 5). **C, D** Representative FACS plots (**C**) and quantification of the absolute number (**D**) of LSK (Lin^−^cKit^+^Sca1^+^) cells, LT-HSC (Lin^−^cKit^+^Sca1^+^Flt3^−^CD150^+^CD48^−^) cells, ST-HSC (Lin^−^cKit^+^Sca1^+^Flt3^−^CD150^−^CD48^−^) cells, MPP4 (Lin^−^cKit^+^Sca1^+^Flt3^+^) cells, MPP3 (Lin^−^cKit^+^Sca1^+^Flt3^−^CD48^+^CD150^−^) cells and MPP2 (Lin^−^cKit^+^Sca1^+^Flt3^−^CD48^+^CD150^+^) cells in WT and *Eva1a*^*F/F,Vav-Cre*^ mice (*n* = 5). **E, F** Representative FACS plots (**E**) and quantification of the absolute number (**F**) of CMP (Lin^−^cKit^+^Sca1^−^CD34^+^CD16/32^−^) cells, GMP (Lin^−^cKit^+^Sca1^−^CD34^+^CD16/32^+^) cells, MEP (Lin^−^cKit^+^Sca1^−^CD34^−^CD16/32^−^) cells and CLP (Lin^−^cKit^mid^Sca1^mid^Flt3^+^IL-7R^+^) cells in WT and *Eva1a*^*F/F,Vav-Cre*^ mice (*n* = 4). Data are presented as mean ± SD, (*p < 0.05, **p < 0.01, and ***p < 0.001).

To investigate the function of EVA1A in HSPCs, we generated two hematopoietic-specific *Eva1a* deletion mice lines: a pIpC-inducible mouse model (*Eva1a*^*flox/flox*^Mx-Cre, hereafter referred to as *Eva1a*^*F/F,Mx-Cre*^) and a genetic knockout mouse model (*Eva1a*^*flox/flox*^Vav-Cre, hereafter referred to as *Eva1a*^*F/F,Vav-Cre*^*)* (Fig. S1A-D). In peripheral blood (PB), *Eva1a* depletion (*Eva1a*^*F/F,Vav-Cre*^) resulted in a significant increase in the frequency of B cells (B220^+^) and a decrease of the T cells (CD4^+^CD8^+^) frequency (Fig. 3A, B). Further analysis showed that compared with the WT mice, the absolute number of the BM cells, LSK cells, LT-HSC (Lin^−^cKit^+^Sca1^+^Flt3^−^CD150^+^CD48^−^) cells, ST-HSC (Lin^−^cKit^+^Sca1^+^Flt3^−^CD150^−^CD48^−^) cells, MPP4 (Lin^−^cKit^+^Sca1^+^Flt3^+^) cells, MPP3 (Lin^−^cKit^+^Sca1^+^Flt3^−^CD48^+^CD150^−^) cells, and MPP2 (Lin^−^cKit^+^Sca1^+^Flt3^−^CD48^+^CD150^+^) cells were increased in *Eva1a*^*F/F,Vav-Cre*^ mice (Fig. 3C, D and Fig. S2A-C). Meanwhile, the number of MEP (Lin^−^cKit^+^Sca1^−^CD34^−^CD16/32^−^) cells, CMP (Lin^−^cKit^+^Sca1^−^CD34^+^CD16/32^−^) cells and GMP (Lin^−^cKit^+^Sca1^−^CD34^+^CD16/32^+^) cells were also dramatically increased in *Eva1a*^*F/F,Vav-Cre*^ mice with the comparable CLP (Lin^−^cKit^mid^Sca1^mid^Flt3^+^IL^−^7R^+^) cells (Fig. 3E, F). These data indicate an increase of HSPCs in *Eva1a* deficient mice.

To further confirm the HSPCs increase induced by *Eva1a* deletion, the absolute number of the HSPCs was also assessed in *Eva1a*^*F/F,Mx-Cre*^ mice with seven injections of pIpC every other day. Consistently, the FACS analysis showed a significant increase in the cell number of the LSK cells, LT-HSC cells, ST-HSC cells, MPP4 cells, MPP3 cells in *Eva1a* deficient mice mediated by Mx-Cre (Fig. S2D). Meanwhile, a dramatically increased number of CMP and GMP cells, but not MEP and CLP cells, were also observed in *Eva1a*^*F/F,Mx-Cre*^ mice (Fig. S2E).

### *Eva1a* deletion enhances the regeneration capacity of HSPCs

To investigate the role of EVA1A in the hematopoietic reconstitution capacity of HSPCs, we conducted competitive transplantation experiments. 1000 LSK cells sorted from WT and *Eva1a*^*F/F,Vav-Cre*^ mice (CD45.2) were respectively mixed with 5 × 10^5^ competitor BM cells (CD45.1) and transplanted into lethally irradiated recipient mice (CD45.1/2). Three months post-transplantation, the donor-derived cells in PB and BM were examined (Fig. S3A). The results showed that same as the donor chimerism of *Eva1a* deficient cells in PB, the donor chimerism of the *Eva1a* deficient LSK, ST (Flt3^−^CD34^+^LSK), MPP (Flt3^+^CD34^+^LSK), and LK (Lin^−^cKit^+^Sca1^−^) cells were dramatically enhanced in the BM of recipient mice as compared with the WT cells (Fig. S3B, C). These data suggest an enhanced regeneration capacity of the *Eva1a* deficient HSPCs.

**Fig. 4:**
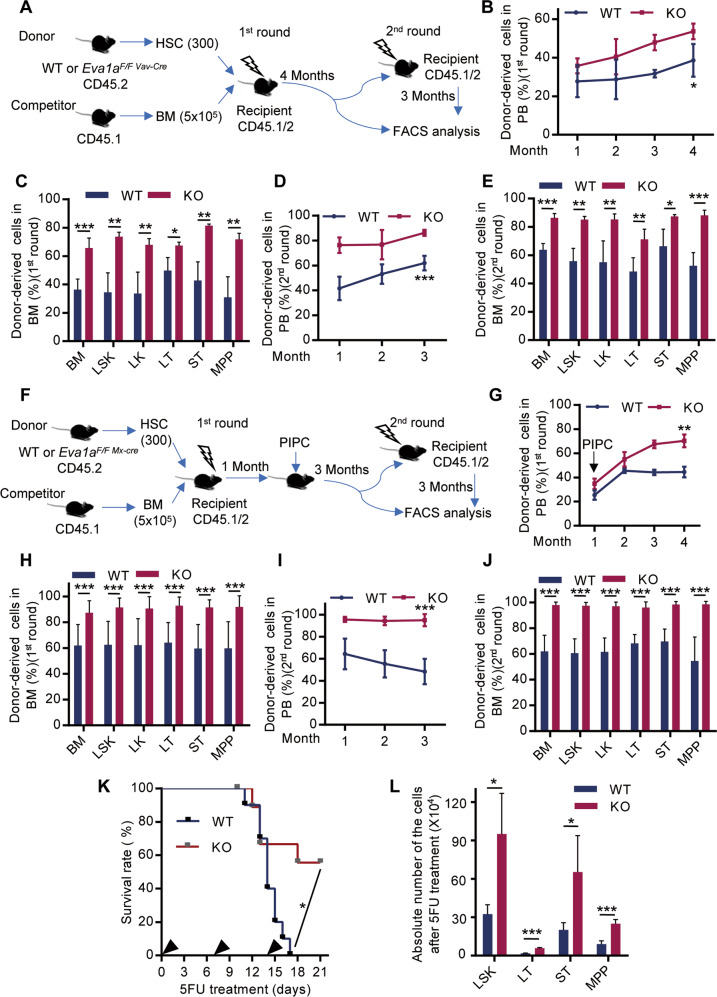
*Eva1a* deletion enhances the regeneration capacity of HSPCs. **A** Experimental schematic for serial competitive transplantation with SLAM-HSC (Lin^−^Sca1^+^cKit^+^CD48^−^CD150^+^) cells from WT and *Eva1a*^*F/F,Vav-Cre*^ mice (results in B-E). **B, D** The percentage of donor-derived cells in PB at the indicated time points during 1st round (**B**) and 2nd round (**D**) of the transplantation (*n* = 5). **C, E** The Percentage of donor-derived BM, LSK (Lin^−^cKit^+^Sca1^+^), LK (Lin^−^cKit^+^Sca1^−^), LT (Flt3^−^CD34^−^LSK), ST (Flt3^−^CD34^+^LSK) and MPP (Flt3^+^CD34^+^LSK) cells in the primary recipients after transplantation 4 months (**C**) and the secondary recipients (**E**) after transplantation 3 months (*n* = 5). **F** Experimental schematic for serial competitive transplantation with SLAM-HSC cells from WT and *Eva1a*^*F/F,Mx-Cre*^ mice (results in **G–J**). **G, I** The percentage of donor-derived cells in PB at the indicated time points during 1st round **(G**) and 2nd round (**I**) of the transplantation (*n* = 9~12). **H, J** The percentage of donor-derived BM, LSK, LK, LT, ST and MPP cells in the primary recipients after transplantation 4 months (**H**) and the secondary recipients (**J**) after transplantation 3 months (*n* = 9~12). **K** Survival curve of WT and *Eva1a*^*F/F,Vav-Cre*^ mice following sequential 5FU treatment. 5FU was injected into mice every week (Black arrow). (*n* = 10 mice per group). **L** Absolute number of the LSK, LT, ST and MPP cells from WT and *Eva1a*^*F/F,Vav-Cre*^ mice at day 8 after 5FU treatment (*n* = 4). Data are presented as mean ± SD, (*p < 0.05, **p < 0.01, and ***p < 0.001).

To further dissect the regeneration capacity of the *Eva1a* deficient HSPCs, we performed serial competitive transplantation experiment, in which the transplanted HSCs experience extensive long-term regenerative stress. 300 SLAM-HSC (Lin^−^cKit^+^Sca1^+^CD48^−^CD150^+^) cells sorted from WT and *Eva1a*^*F/F,Vav-Cre*^ mice (CD45.2) were respectively mixed with 5 × 10^5^ competitor BM cells (CD45.1) and transplanted into lethally irradiated recipient mice (CD45.1/2), and four months later, the total BM cells were isolated from the primary recipients, and transplanted into the secondary recipients. The donor-derived cells in PB and BM were examined at the first and second round of transplantation (Fig. 4A). The results showed that compared with the WT cells, *Eva1a* depletion significantly enhanced the donor chimerism of the BM, LSK, LK, LT, ST, and MPP cells in the BM of recipient mice, and the percentage of donor-derived cells in PB of recipient mice after both the first and second transplantation (Fig. 4B-E). Meanwhile, the same serial competitive transplantation experiment was performed by the donor-derived HSCs from the pIpC-induced *Eva1a* deficient mice (*Eva1a*^*F/F,Mx-Cre*^). And a similar promotion of the regeneration capacity was observed in *Eva1a* deficient HSCs from pIpC-induced *Eva1a* deficient mice (Fig. S3D-H). These data indicate that *Eva1a* deletion promotes the regeneration capacity of the HSCs. In addition, to further investigate whether the regeneration capacity promotion of *Eva1a* deficient HSCs was due to the engraftment of the HSCs, the WT and *Eva1a*^*F/F,Mx-Cre*^ HSCs were first transplanted without inducing *Eva1a* deletion. Then the recipient mice were injected with pIpC at 1 month after transplantation to induce *Eva1a* deletion, followed by PB chimerism analysis and secondary transplantation (Fig. 4F). The results showed *Eva1a* deletion similarly enhanced the regeneration capacity of HSCs after both the first and second transplantation (Fig. 4G-J). Those data confirmed that *Eva1a* deletion enhanced the regeneration capacity of the HSCs in a cell-intrinsic manner.

Lastly, to examine the regeneration capacity of *Eva1a* deficient HSPCs after hematopoietic ablation, *Eva1a* deficient mice were treated with 5FU, the number of the HSPCs and the survival rate of the mice were assessed. *Eva1a* deletion significantly enhanced the survival rate of the mice treated with 5FU (Fig. 4K). Meanwhile, the *Eva1a* deficient mice have more LSK, LT, ST, and MPP cells after 5FU treatment 8 days (Fig. 4L). Taken together, these data indicate an enhanced regeneration capacity of the *Eva1a* deficient HSPCs.

### *Eva1a* deficiency does not affect HSPC proliferation and autophagy

Previous study indicates that EVA1A is involved in autophagosome formation [17]. To investigate the mechanism underlying the enhanced regeneration capacity of the *Eva1a* deficient HSPCs, we first analyzed the effect of *Eva1a* deletion on the HSPC autophagosome formation. The results showed that *Eva1a* deletion did not affect the autophagosome formation of the HSPCs as demonstrated by the comparable conversion of LC3I/II between WT and *Eva1a* deficient Lin^−^ BM cells, even after 5FU treatment and transplantation (Fig. S4A, C, E). In addition, the autophagic degradation was also measured in the same cells. The results showed no significant difference in the autophagic degradation protein levels, such as P62, Binp3, TOM20 and TIM23 between WT and *Eva1a* deficient Lin^−^ BM cells, even after 5FU treatment and transplantation (Fig. S4B, D, F). Taken together, these data suggest that *Eva1a* deletion does not affect HSPCs autophagy.

Given the increase of HSPCs number in *Eva1a* deficient mice and the enhanced regeneration capacity of HSPCs induced by *Eva1a* deletion, we examined the proliferation of the *Eva1a* deficient HSPCs. Ki67 staining showed a comparable frequency of cycling SLAM-HSCs between WT and *Eva1a* deficient mice (Fig. S5A). Bromodeoxyuridine (BrdU) labeling further confirmed the comparable proliferation of the WT and *Eva1a* deficient SLAM-HSCs (Fig. S5B). Cycling hematopoietic cells are more sensitive to 5FU cytotoxicity [18]. To further confirm the proliferation of *Eva1a* deficient HSPCs, we assessed the proliferation of *Eva1a* deficient HSPCs under 5FU treatment. The Ki67 staining showed a comparable proliferation of the WT and *Eva1a* deficient LSK after 5FU treatment (Fig. S5C). Lastly, the equivalent proliferation was also observed after transplantation in *Eva1a* knockout LSK cells (Fig. S5D). Taken together, these data suggest that *Eva1a* deficiency does not affect HSPCs proliferation.

### *Eva1a* deletion reduces HSPC apoptosis during hematopoietic regeneration

**Fig. 5:**
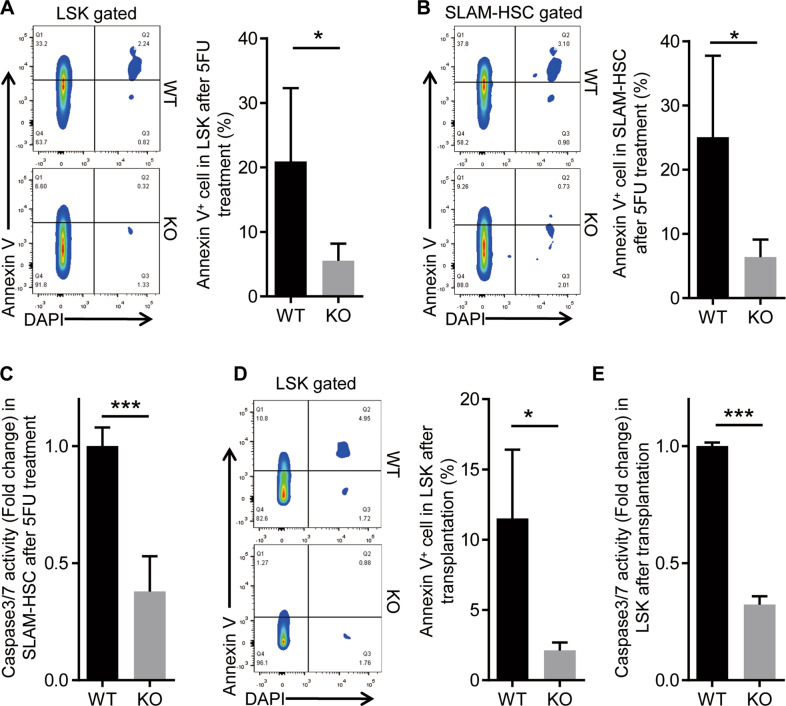
*Eva1a* deletion reduces HSPCs apoptosis during hematopoietic regeneration. **A, B** Representative FACS plots (left) and the percentage (right) of Annexin V positive LSK (Lin^−^cKit^+^Sca1^+^) cells (**A**) and SLAM-HSC (Lin^−^Sca1^+^cKit^+^CD48^−^CD150^+^) cells (**B**) in WT and *Eva1a*^*F/F,Vav-Cre*^ mice after 5FU treatment 8 days (*n* = 5). **C** Caspase 3/7 activity of the SLAM-HSC cells from WT and *Eva1a*^*F/F,Vav-Cre*^ mice after 5FU treatment 8 days (*n* = 8). **D** CD45.1 mice after lethal irradiation were transplanted with 1 × 10^6^ BM cells from WT and *Eva1a*^*F/F,Vav-Cre*^ mice for 14 days. Representative FACS plots (left) and bar graph (right) depict the percentage of Annexin V positive cells in donor-derived LSK cells (*n* = 5). **E** Caspase 3/7 activity of the LSK cells as in (**D**) (*n* = 3). Data are presented as mean ± SD, (*p < 0.05, and ***p < 0.001).

To further unravel the mechanism underlying the enhanced regeneration capacity of the *Eva1a* deficient HSPCs, we analyzed the HSPCs apoptosis during hematopoietic regeneration. The results showed compared with the WT mice, a significantly reduced frequency of Annexin V positive cells in LSK and SLAM-HSCs of the *Eva1a* deficient mice after 5FU treatment (Fig. 5A, B). Caspase 3/7 activity assay further confirmed a lower apoptosis in *Eva1a* deficient SLAM-HSCs after 5FU treatment than that in WT cells (Fig. 5C). Meanwhile, the apoptosis rate was also assessed in the WT and *Eva1a* deficient HSPCs after transplantation. Annexin V staining analysis indicated a significantly lower apoptosis in *Eva1a* deficient LSK cells after transplantation than that in WT cells (Fig. 5D). Consistently, compared to the WT cells, a dramatically lower caspase 3/7 activity was detected after transplantation in *Eva1a* deficient LSK cells (Fig. 5E). Taken together, these data suggest that *Eva1a* deletion reduces HSPCs apoptosis during hematopoietic regeneration.

### ER stress-induced EVA1A and MCL1 interaction stimulates mitochondria-associated apoptosis

**Fig. 6:**
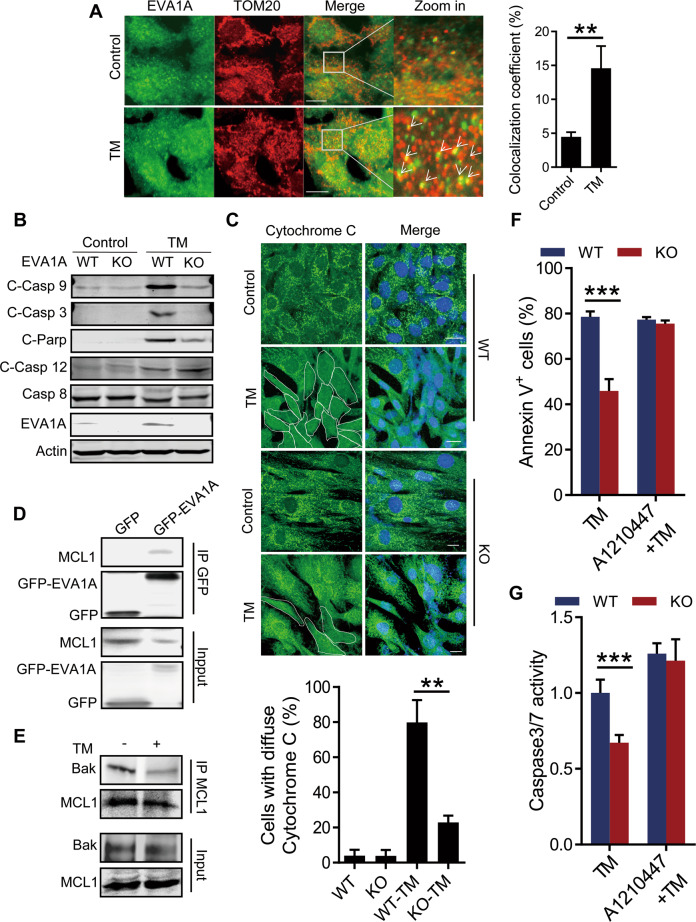
ER stress-induced EVA1A and MCL1 interaction stimulates mitochondria-associated apoptosis. **A** Representative images (left) and quantification (right) showing the colocalization of EVA1A and TOM20 in MEF cells treated without or with 3 μg/ml Tunicamycin (TM) for 48 h. The colocalization coefficient was represented as percentage of signals of EVA1A that were positive for TOM20. Quantifications were performed using Volocity software (*n* > 20 cells). Scale bar, 10 μm. **B** Western blot analysis of the cleaved Caspase 9, cleaved Caspase 3, cleaved Caspase 12, cleaved PARP and Caspase 8 proteins level in WT and *Eva1a* knockout MEF cells treated without or with 3 μg/ml TM for 48 h. **C** Representative images (top) and quantification (bottom) showing cytochrome C localization in WT and *Eva1a* knockout MEF cells treated without or with TM for 24 h (*n* > 35 cells). Scale bar, 20 μm. **D** HEK293 cells expressing GFP or GFP-EVA1A were treated with 3 μg/ml TM for 48 h and immunoprecipitated with anti-GFP antibody, then the immunocomplexes were analyzed by Western blot. **E** HEK293 cells were treated without or with TM and immunoprecipitated with anti-MCL1 antibody, then the immunocomplexes were analyzed by Western blot. **F** MEF cells were treated with TM, or TM + A1210447 for 36 h. Bar graph depicts the percentage of Annexin V positive cells (*n* = 3). **G** Caspase 3/7 activity of the MEF cells treated as in (**F**) (*n* = 5). Data are presented as mean ± SD, (**p < 0.01, and ***p < 0.001).

To investigate the molecular mechanism underlying the apoptosis inhibition of the *Eva1a* deficient HSPCs, we first analyzed the localization of EVA1A under ER stress. Interestingly, a significantly mitochondrial localization of EVA1A was observed in MEF cells after tunicamycin treatment as demonstrated by the colocalization of EVA1A with TOM20, a classical mitochondrial marker (Fig. 6A). And overexpressing GFP-EVA1A in HEK293 cells induced a significant mitochondria-associated apoptosis as demonstrated by the enhanced caspase 3/7/9 activity, Annexin V positive cells and the decreased mitochondrial membrane potential (Fig. S6A-D). These data suggested a role of EVA1A in mitochondria-associated apoptosis. To confirm this, we further checked the protein levels of caspase under ER stress. The results showed compared to the WT cells, *Eva1a* deletion significantly inhibited the protein levels of cleaved caspase 3 and Parp accompanying by a comparable caspase 8 and cleaved caspase 12 in MEF cells treated with or without tunicamycin (Fig. 6B). Notably, a significant reduction of cleaved caspase 9, a caspase involved in mitochondria-associated apoptosis, was observed in *Eva1a* deficient MEF cells treated with tunicamycin (Fig. 6B). And *Eva1a* deletion significantly reduced the cytochrome C release from mitochondria as demonstrated by a decreased localization of the diffuse cytochrome C in Eva1a deficient MEF cells after tunicamycin treatment (Fig. 6C and Fig. S7A, B). Meanwhile, *Eva1a* deletion dramatically inhibited the fragmentation of mitochondrial after tunicamycin treatment (Fig. S7C). These data indicate an inhibited mitochondria-associated apoptosis in *Eva1a* deficient cells.

To further investigate the molecular mechanism underlying the inhibited mitochondria-associated apoptosis in *Eva1a* deficient cells, we dissected the interaction of EVA1A with a series of mitochondrial proteins. Interestingly, a significant interaction of EVA1A with MCL1, a protein involved in mitochondria-associated apoptosis regulation, was detected in cells after tunicamycin treatment accompanying by a decreased interaction of MCL1 with Bak (Fig. 6D, E). And this decreased interaction of MCL1 with Bak was abolished after *Eva1a* deletion (Fig. S6E). These data suggest a function of EVA1A on apoptosis by interacting with MCL1 and impairing the interaction of MCL1 with Bak. Lastly, the function of EVA1A on apoptosis via MCL1 was further confirmed by treating the cells with MCL1 inhibitor. Both the Annexin V staining and caspase 3/7 activity analysis showed MCL1 inhibitor A1210447 treatment impaired the apoptosis inhibition in *Eva1a* deficient MEF cells after tunicamycin treatment (Fig. 6F, G). Taken together, these data suggest during ER stress, EVA1A stimulated a mitochondria-associated apoptosis via inducing the interaction of EVA1A and MCL1.

### *Eva1a* deficiency inhibits the mitochondria-associated apoptosis in HSPCs via MCL1 during ER stress

**Fig. 7:**
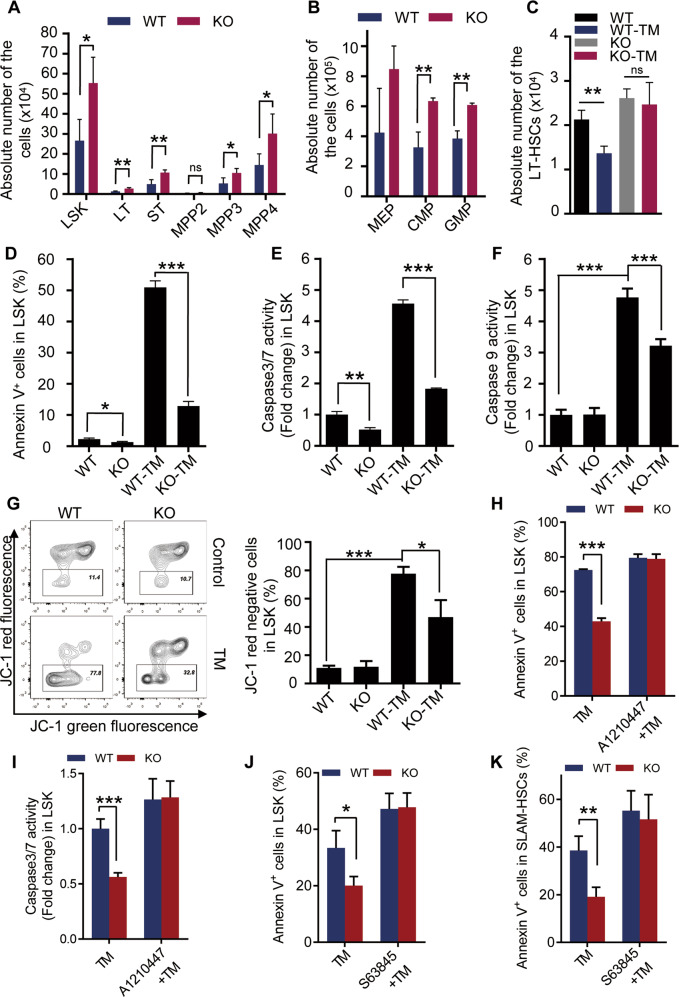
*Eva1a* deficiency inhibits ER stress-induced apoptosis in HSPCs via MCL1. **A–C** WT and *Eva1a*^*F/F,Vav-Cre*^ mice were intraperitoneally injected with 0.5 mg/kg Tunicamycin (TM) every 3 days. **A** The absolute number of the LSK, LT-HSC, ST-HSC, MPP2, MMP3, and MPP4 cells in the TM treated mice were measured at 7th day (*n* = 4). **B** The absolute number of CMP, GMP and MEP cells in the mice as in (**A**) (*n* = 3). **C** The absolute number of the LT-HSCs in the mice treated with or without TM at 7th day (*n* = 3). **D** LSK cells were sorted from WT and *Eva1a*^*F/F,Vav-Cre*^ mice by flow cytometry, and in vitro-treated with or without 3 μg/ml TM for 36 h in SFEM medium containing 50 ng/ml SCF and 50 ng/ml TPO. Bar graph depict the percentage of Annexin V positive cells (*n* = 3). **E** Caspase 3/7 activity of the LSK cells as in (**D**) (*n* = 3). **F** Caspase 9 activity of the WT and *Eva1a* knockout LSK cells treated with or without 3 μg/ml TM for 36 h in vitro (*n* = 3–5). **G** LSK cells from WT and *Eva1a*^*F/F,Vav-Cre*^ mice were treated with or without 3 μg/ml TM for 36 h in vitro. Representative FACS plots (left) and bar graph (right) depict the percentage of JC-1 red fluorescence negative cells (*n* = 3). **H** LSK cells from WT and *Eva1a*^*F/F,Vav-Cre*^ mice were treated with TM or TM + A1210447 for 36 h in vitro. Bar graph depicts the percentage of Annexin V positive cells (*n* = 5). **I** Caspase 3/7 activity of the WT and *Eva1a* knockout LSK cells treated as in (**H**). (*n* = 5). **J, K** WT and *Eva1a*^*F/F,Vav-Cre*^ mice were intraperitoneally injected with TM or TM + S63458 every 3 days. The percentage of Annexin V positive LSK (J) and SLAM-HSC (K) cells were measured at 7th day (*n* = 4~5). Data are presented as mean ± SD, (*p < 0.05, **p < 0.01, and ***p < 0.001).

ER stress plays an important role in HSPC regulation [10–12]. In this study, our data indicate that the EVA1A expression was stimulated by ER stress during hematopoietic regeneration (Fig. 2), and *Eva1a* deletion reduces HSPCs apoptosis during hematopoietic regeneration (Fig. 5). These data suggest an important role of ER stress in the regeneration capacity of HSPCs regulated by EVA1A. To test this hypothesis, the number of HSPCs was also assessed in *Eva1a* deficient mice treated with tunicamycin, a classical ER stress inducer. The FACS analysis showed that after tunicamycin treatment, *Eva1a* deficient mice had more LSK, LT-HSC, ST-HSC, MPP3, MPP4, CMP, and GMP cells in BM than that in WT mice (Fig. 7A, B). *Eva1a* deletion significantly alleviated the number reduction of LT-HSC cells in the mice treated with tunicamycin (Fig. 7C). In addition, the apoptosis rate was assessed in the WT and *Eva1a* deficient HSPCs after tunicamycin treatment in vitro. Annexin V staining analysis indicated a significantly lower apoptosis in *Eva1a* deficient LSK cells after tunicamycin treatment than that in WT cells (Fig. 7D). Consistently, compared to the WT cells, a dramatically lower caspase 3/7 activity was observed in *Eva1a* deficient LSK cells after tunicamycin treatment in vitro (Fig. 7E). These data indicate *Eva1a* deficiency inhibits ER stress-induced apoptosis in HSPCs.

Given ER stress-induced EVA1A and MCL1 interaction stimulates mitochondria-associated apoptosis in MEF cell, we also examined the mitochondria-associated apoptosis in HSPCs under ER stress. Consistently, compared to the WT cells, a dramatically lower caspase 9 activity was observed in *Eva1a* deficient LSK cells after tunicamycin treatment (Fig. 7F). Meanwhile, a higher mitochondrial membrane potential was detected in *Eva1a* deficient LSK cells after tunicamycin treatment than that in WT LSK cells (Fig. 7G). These data indicate an inhibited mitochondria-associated apoptosis in *Eva1a* deficient HSPCs during ER stress.

Lastly, the function of EVA1A on apoptosis via MCL1 was further confirmed in HSPCs by treating the HSPCs with MCL1 inhibitor in vitro and *vivo*. Both the Annexin V staining and caspase 3/7 activity analysis showed MCL1 inhibitor A1210447 treatment impaired the apoptosis inhibition in *Eva1a* deficient LSK cells after tunicamycin treatment in vitro (Fig. 7H, I). And a similar phenomenon was also detected in the LSK and SLAM-HSC cells from WT and *Eva1a* deficient mice treated with tunicamycin or tunicamycin and S63845 (Fig. 7J, K), another MCL1 inhibitor which shows no cytotoxicity to the hemopoietic system [19]. Taken together, these data suggest *Eva1a* deficiency inhibits the mitochondria-associated apoptosis in HSPCs via MCL1 during ER stress.

## Discussion

In this study, we report a previously unknown function of EVA1A protein in HSPCs homeostasis and regeneration (Fig. S9). Our results indicate that EVA1A is an ER stress response protein and involves in ER-mitochondria-mediated apoptosis by interacting with MCL1. Deletion *Eva1a* significantly enhances the regeneration capacity of HSPCs.

Under ER stress, the fate of the cell is determined by the intensity and duration of the stress and the context of cell. The UPR is firstly activated as an adaptive response to restore the balance of ER homeostasis by restricting new protein synthesis and promoting protein folding and degradation, and apoptosis will be induced after persistent or strong ER stress. In this study, we found that hematopoietic regeneration induces a significant ER stress of HSPCs. *Eva1a* deletion inhibits the ER stress-induced apoptosis and enhances the regeneration capacity of HSPCs during hematopoietic regeneration. These data are consistent with previous reports that the suppression of ER stress-associated apoptosis enhances HSC reconstitution capacity [11, 12]. It is reported that compared to their progenitors, HSCs are more predisposed to apoptosis under ER stress [5]. Consistently, our data show a higher apoptosis in SLAM-HSCs compared to the LSK cells under tunicamycin-induced ER stress (Fig. 7J, K). In addition, compared to the WT mice, an enhanced number of the HSCs and their progenitors were both observed in *Eva1a* deficient mice after tunicamycin treatment (Fig. 7A, B). These data suggest a significant function of EVA1A on the ER stress-induced hematopoietic stem and progenitor cells apoptosis, which finally enhanced the hematopoietic stem and progenitor cell maintenance and the hematopoietic reconstitution capacity of the *Eva1a* deficient mice. Recently, the UPR signaling pathway, such as IRE1α–XBP1 was reported to preserve the self-renewal of HSCs under ER stress [10], suggesting the proper management of ER stress signaling is essential for maintaining HSCs homeostasis. In this study, besides the inhibition of apoptosis induced by *Eva1a* deletion during hematopoietic regeneration, a mild expression increases of the UPR target gene, such as *Bip* and *Xbp1-s*, but not *Chop* were also observed in *Eva1a* deficient HSPCs under steady state (Fig. S8). These data suggest *Eva1a* deletion may lead to a mild UPR to clean the damaged proteins, which may play a role in the increased HSPCs in *Eva1a* deficient mice under steady state. One of the most likely reasons is the mild ER stress may increase the threshold of the ER stress-induced HSC impairment by enhancing the chaperone protein such as Bip, and upregulate the efficiency of HSC unfolded protein response. However, how the *Eva1a* deficiency leads to the mild UPR under HSC steady state is not deeply investigated in this study. It will be interesting to further clarify the mechanism in future study.

CHOP is known as a key mediator of ER stress-induced apoptosis, but the molecular mechanism remains far from being completely understood. Previous studies show CHOP regulates apoptosis by transcriptionally regulating Bcl-2 family proteins such as BIM, BCL2, and PUMA [20–22]. However, other work showed a role of CHOP in apoptosis by transcriptionally increasing protein synthesis leading to cell death without any change of the Bcl-2 family proteins [23]. These works suggest a different mechanism of CHOP in apoptosis under different cell types or contexts. In this study, we identified a novel function of CHOP that upregulated the expression of EVA1A protein, which promoted the ER-mitochondria-mediated apoptosis by interacting with MCL1. Meanwhile, our data show *Eva1a* deficiency enhances the regeneration capacity of HSPCs via reducing apoptosis, which is consistent with our previous study showing *Chop* deletion improves HSCs regeneration through decreasing apoptosis [16]. EVA1A is an ER membrane protein involved in autophagy and apoptosis [13]. To date, the molecular mechanism of the EVA1A-induced apoptosis is still unknown. In this study, we found that ER stress induced a CHOP-dependent expression of EVA1A which interacted with MCL1, an anti-apoptotic Bcl-2 family protein required for HSC survival [24], leading to a drop in mitochondrial membrane potential and the release of cytochrome C from mitochondria which finally induced apoptosis. Regrettably, although a potential role of EVA1A in autophagosome formation was reported [17], a comparable autophagy was observed in WT and *Eva1a* deletion HSPCs in this study. However, it is possible that the EVA1A-involved autophagy play a role in the tissue with high metabolic activity, such as liver and heart [25, 26] or contexts under dramatical metabolic fluctuations, such as starvation [27].

MCL1 is an essential regulator of HSC self-renewal and survival [24, 28]. In this study, our data show a significant binding of EVA1A with MCL1 leading a reduction of MCL1 and Bak interaction. *Eva1a* deletion significantly inhibited the HSPC apoptosis during hematopoietic regeneration confirming the important role of the MCL1 in HSCs. In addition, MCL1 is required for the mitochondrial homeostasis. MCL1 deletion results in mitochondria disassemble, cristae morphological abnormalities and defects in electron-transport-chain enzymatic function [29]. Given the significant interaction of EVA1A and MCL1, a potential role of the mitochondrial homeostasis in the *Eva1a* deficient HSC will be interesting in the future study. In addition, we found a significant increase of B cells, and a decrease of T cells in the PB of *Eva1a* deficient mice under steady state. But *Eva1a* deletion did not change the number of CLP cells in BM. Given the important role of apoptosis in the development and activity of T and B cells [30], a potential role of the EVA1A in the differentiation or maturation of T and B cells will be interesting in future study.

## Materials And Methods

### Mice

*Eva1a*^flox/flox^ (hereafter referred to as *Eva1a*^*F/F*^) mice were kindly provided by Dr. Chen Yingyu [14]. To obtain tissue-specific or inducible *Eva1a* knockout mice, the *Eva1a*^*F/F*^ mice were crossed with Vav-Cre, Mx-Cre and ERT-Cre transgenic mice. *Chop* knockout mice was used as described previously [16]. B6.SJL-PtprcaPep3b/Boy (CD45.1) mice or CD45.1/CD45.2 heterozygous mice were used as recipients or competitors for HSCs transplantation [31]. All of these strains were maintained in a C57BL/6 background and were housed in a specific pathogen-free environment. The Animal Care and Ethics Committee of Jinan University approved all animal experiments in this study.

### Cell culture, transfection and knockdown

MEF, Huh7 and HEK293 cells were grown in DMEM with 10% FBS at 37 °C under 5% CO_2_. No mycoplasma contamination was detected. Transient transfection was performed by Lipofectamine 3000/2000 (Invitrogen) according to the manufacturer’s instructions. For the IRE1 and ATF6 knockdown, siRNA duplexes designed against conserved targeting sequences were transfected into cells at a final concentration of 20 nM using Lipofectamine 2000 as specified by the manufacturer. The following siRNA duplexes were used: 5′-GCUGAACUACUUGAGGAAUUA-3′ for IRE1; 5′-GGCAAAGCAGCAGUCGAUUAU-3′ for ATF6. For liquid culture, HSPCs were cultured in serum-free expansion medium (SFEM) (Stem cell Technologies) with 50 ng/ml stem cell factor (Pepro Tech), and 100 U/ml penicillin/streptomycin.

### Flow cytometry and cell sorting

Bone marrow (BM) cells from femurs, tibiae, and iliac crests were isolated as reported previously [31]. Before cell sorting, BM cells were first enriched with anti-antigen-presenting cell microbeads (MiltenyiBiotec) and then stained with surface markers. Prepared samples were analyzed on the LSR Fortessa^TM^ cell analyzer (BD Biosciences) or sorted on the FACS AriaIII cell sorter (BD Biosciences). Peripheral blood from the postorbital vein was collected and analyzed on LSR Fortessa^TM^ cell analyzer (BD Biosciences). Detailed methods and antibodies were as our lab paper described previously [31].

### Chemical Treatment

For 5FU (Sigma) treatment, mice were intraperitoneally injected 5FU at 150 mg/kg and sacrificed at the indicated time point. For 5FU treatment survival assay, the mice were persistently and intraperitoneally injected 5FU at 150 mg/kg every week. For the pIpC (Sigma) treatment, mice received 20 mg/kg every other day for 2 weeks. For the tunicamycin treatment, the mice were intraperitoneally injected 0.5 mg/kg tunicamycin every 3 days and sacrificed at the indicated time point. For S63845 (APExBIO) treatment, mice were intraperitoneally injected S63845 at 10 mg/kg every 3 days and sacrificed at the indicated time point. For the in vitro experiments, HSPCs were sorted and cultured in SFEM (Stem cell Technologies) with 50 ng/ml SCF and 50 ng/ml TPO, then were treated with 3 μg/ml tunicamycin or 10 μM A1210447 as indicated in the figure legends. Cell lines were cultured in DMEM with 10% FBS, and treated with 3 μg/ml tunicamycin or 0.2 μM thapsigarginas indicated in the figure legends.

### Immunoprecipitation and western blot

For immunoprecipitation, cells were lysed with NP40 lysis buffer (50 mM Tris-HCl pH 7.5, 100 mM NaCl, 1% NP-40, 1 mM EDTA, 1 mM DTT, 10% glycerol) containing protease inhibitors. After centrifugation, the supernatants were incubated with antibody overnight and then Protein A/G agarose for 2 h at 4 °C. Immunocomplexes were washed and analyzed by Western blot. For Western blot, the proteins from lysed cells were denatured and separated with SDS-PAGE. Then, the proteins were transferred to PVDF membranes, blocked and incubated with the corresponding primary and secondary antibodies. The specific bands were analyzed by the Western blot infrared imaging system (LI-COR Biosciences). The following antibodies were used: EVA1A: A8070, ABclonal; CHOP: 2895, Cell signaling Technology (CST); IRE1: 14C10, CST; ATF6: 65880 T, CST; Actin: AC026, ABclonal; Flag: 20543-1-AP, Proteintech; Caspase 8: 4927, CST; C-Caspase 9: 9509, CST; C-Caspase 3: 9664, CST; Caspase 12: 2202, CST; C-Parp: 9548, CST; GFP: ab290, Abcam; MCL1: 94296, CST; Bak: 12105, CST; LC3: 27543,Sigma; P62: 18420-1-AP, Proteintech; BNIP3: ab10433, Abcam; TIM23: 111263-A, Proteintech; TOM20: ab56783, Abcam.

### Immunostaining and confocal microscopy

For immunostaining, cells were fixed in 4% formaldehyde in PBS for 10 minutes at room temperature. After washing with PBS, they were incubated in PBS containing 10% FCS to block nonspecific sites of antibody adsorption. Then, the cells were incubated with appropriate primary and secondary antibodies in 0.1% saponin as indicated in the figure legends. Images were taken in multi-tracking mode on a laser scanning confocal microscope (LSM880, Carl Zeiss) with a 63х plan apochromat 1.4 NA objective. The following antibodies were used: EVA1A: NBP1-92517, NOVUS; TOM20: ab56783, Abcam; Cytochrome C: ab133504, Abcam.

### RNA isolation and Real-time PCR Analysis

Total RNA was isolated from the cells using RNeasy Micro Kit (Qiagen) according to the manufacturer’s instructions. RNA was reversed by the PrimeScript cDNA synthesis Kit (Takara). The quantitative-PCR was performed on a 7300 Real-Time PCR system for 40 cycles. The expression of β-actin was used as the internal control. At least three biological replicates were performed for each experiment.

### Apoptosis and cell cycle assays

For apoptosis assay, BM cells were stained with antibodies for the surface markers, and incubated with the Annexin V-FITC antibody at room temperature for 15 minutes. DAPI (1 μg/ml) was added before analysis by FACS. For the in vitro apoptosis assay, the cells treated with the drug in vitro were directly stained with the Annexin V-FITC antibody, and DAPI was added before analysis by FACS. For caspase 3/7, 9 activity assays, HSPCs were plated into 96-well white luminescence plates and the Caspase3/7, 9 activities were analyzed according to the manufacturer’s instructions (Promega). For the mitochondrial membrane potential assay, the cells treated with the drug in vitro were determined by FACS after loading the cells with JC-1 dye according to the manufacturer’s instructions (Beyotime). For the BrdU incorporation assay, BrdU (100 mg/kg) was intraperitoneally injected, and followed by administration of BrdU (1 mg/ml) in the drinking water for 10 days. BrdU incorporation was determined by FACS analysis using the BrdU Flow Kit (BD Biosciences). For Ki67 staining, after the surface markers staining, the cells were fixed using BrdU Flow Kit and staining with Ki67 antibody (BD Pharmingen).

### Transplantation analysis

Competitive transplantation was performed by transplanting 300 SLAM-HSCs (CD150^+^CD48^−^Lin^−^Sca1^+^cKit^+^) sorted from donor mice (CD45.2) with 5 × 10^5^ BM cells from age-matched WT mice (competitor, CD45.1) into lethally irradiated recipient mice (CD45.1/CD45.2). For the secondary transplantation, 1 × 10^6^ chimeric BM cells from the primary recipients were transplanted into the secondary recipient mice at 4th month after primary transplantation. Peripheral blood cells from recipients were analyzed over time. Donor-derived bone marrow cells were collected from recipient mice at indicated time point after transplantation.

### Protein aggregation analysis

Cells were fixed and permeabilized by BD Fixation/Permeabilization Solution kit (BD Biosciences). The protein aggregation level in the cells were analyzed by FACS using ProteoStat Dye (Enzo Life Sciences).

### Statistical analyses

The sample sizes were described in the figure legend and were determined as at least three biologically independent animals according to previous studies performed by our group. Animals with the same genotype and gender and similar age (3~4 months) were randomly assigned to experimental groups. Investigators were not blinded during the group allocation during the experiment. All statistical analyses were performed with GraphPad Prism 7 software. Data are presented as the mean ± SD. The statistical significance of the differences between groups was calculated using the unpaired Student’s two-tailed t-test. The survival curve was analyzed by using a log-rank (Mantel-Cox) test. The significance level was set at 0.05. *p < 0.05, **p < 0.01, ***p < 0.001.

## Supplementary information

Supplemental Figure

Original Data File

reproducibility checklist

## Data Availability

The datasets supporting the conclusions of this article are included within the article and its Additional files.
