# Supplementary material for: EVA1A regulates hematopoietic stem cell regeneration via ER-mitochondria mediated apoptosis
Source: Cell Death Dis. 2023 Jan 30;14(1):71. doi: 10.1038/s41419-023-05559-9 (PMC9887066; doi:10.1038/s41419-023-05559-9)
Supplement: Supplementary file 1 — Supplemental Figure [file 41419_2023_5559_MOESM1_ESM.pdf]

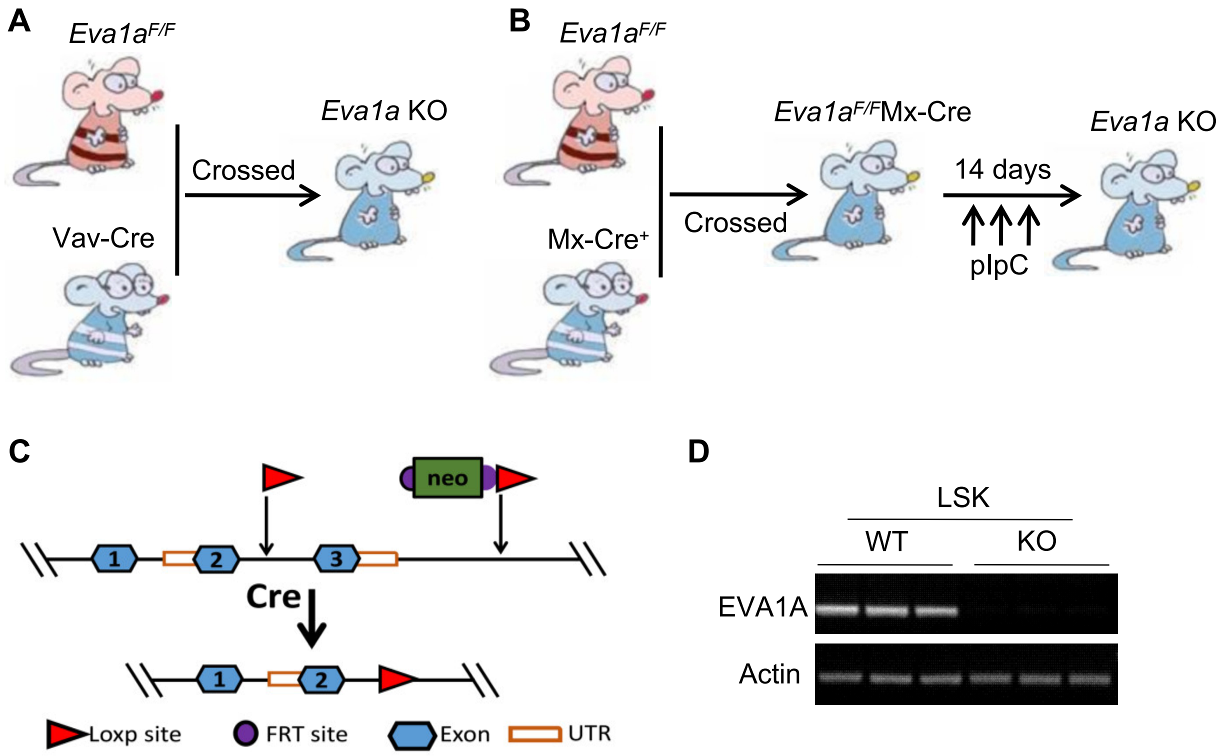

Figure S1. Generation of *Eva1a* Knockout Mice. (A, B) Two hematopoietic-specific *Eva1a* deletion mice lines were generated by crossing *Eva1a*$^{F/F}$ mice with Vav-Cre transgenic mice to generate a genetic *Eva1a* knockout mouse (A) or Mx-Cre transgenic mice to generate a pIpC-inducible *Eva1a* deletion mice (B). (C) Experimental schematic for the generation of mice with HSC-specific deletion of *Eva1a*. (D) The deficiency of *Eva1a* was checked by RT-PCR in the LSK (Lin^{-} Sca1^{+} cKit^{+}) cells from WT and *Eva1a* knockout mouse.

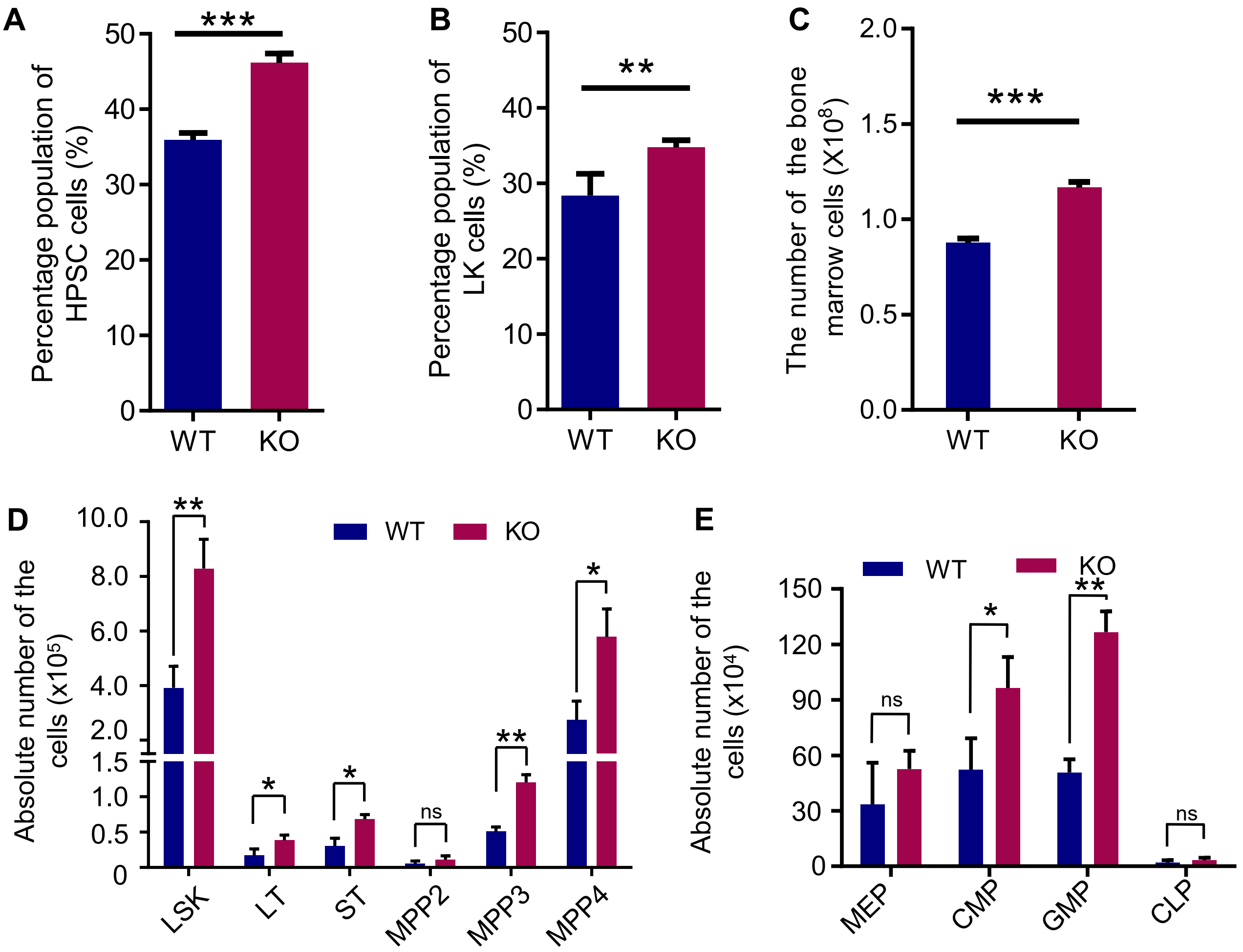

Figure S2. *Eva1a* deficiency impairs HSCs homeostasis. (A, B) The percentage population of the HPSC (Lin$^-$cKit$^+$Sca1$^+$Flt3$^-$) and LK (Lin$^-$cKit$^+$Sca1$^-$) cells in WT and *Eva1a*$^{F/F,Vav\text{-}Cre}$ mice (n=4~5). (C) The absolute number of the bone marrow cells in WT and *Eva1a*$^{F/F,Vav\text{-}Cre}$ mice (n=5). (D) The absolute number of LSK (Lin$^-$cKit$^+$Sca1$^+$) cells, LT-HSC (Lin$^-$cKit$^+$Sca1$^+$Flt3$^-$CD150$^+$CD48$^-$), ST-HSC (Lin$^-$cKit$^+$Sca1$^+$Flt3$^-$CD150$^-$CD48$^-$) cells, MPP4 (Lin$^-$cKit$^+$Sca1$^+$Flt3$^+$) cells, MPP3 (Lin$^-$cKit$^+$Sca1$^+$Flt3$^-$CD48$^+$CD150$^-$) cells and MPP2 (Lin$^-$cKit$^+$Sca1$^+$Flt3$^-$CD48$^+$CD150$^+$) cells in WT and *Eva1a*$^{F/F,Mx\text{-}Cre}$ mice (n=3). (E) The absolute number of CMP (Lin$^-$cKit$^+$Sca1$^-$CD34$^+$CD16/32$^-$) cells, GMP (Lin$^-$cKit$^+$Sca1$^-$CD34$^+$CD16/32$^+$) cells, MEP (Lin$^-$cKit$^+$Sca1$^-$CD34$^-$CD16/32$^-$) cells and CLP (Lin$^-$cKit^{mid}Sca1^{mid}Flt3$^+$IL-7R$^+$) cells in WT and *Eva1a*$^{F/F,Mx\text{-}Cre}$ mice (n=3). Data are presented as mean ± SD, (*$p < 0.05$, **$p < 0.01$ and ***$p < 0.001$).

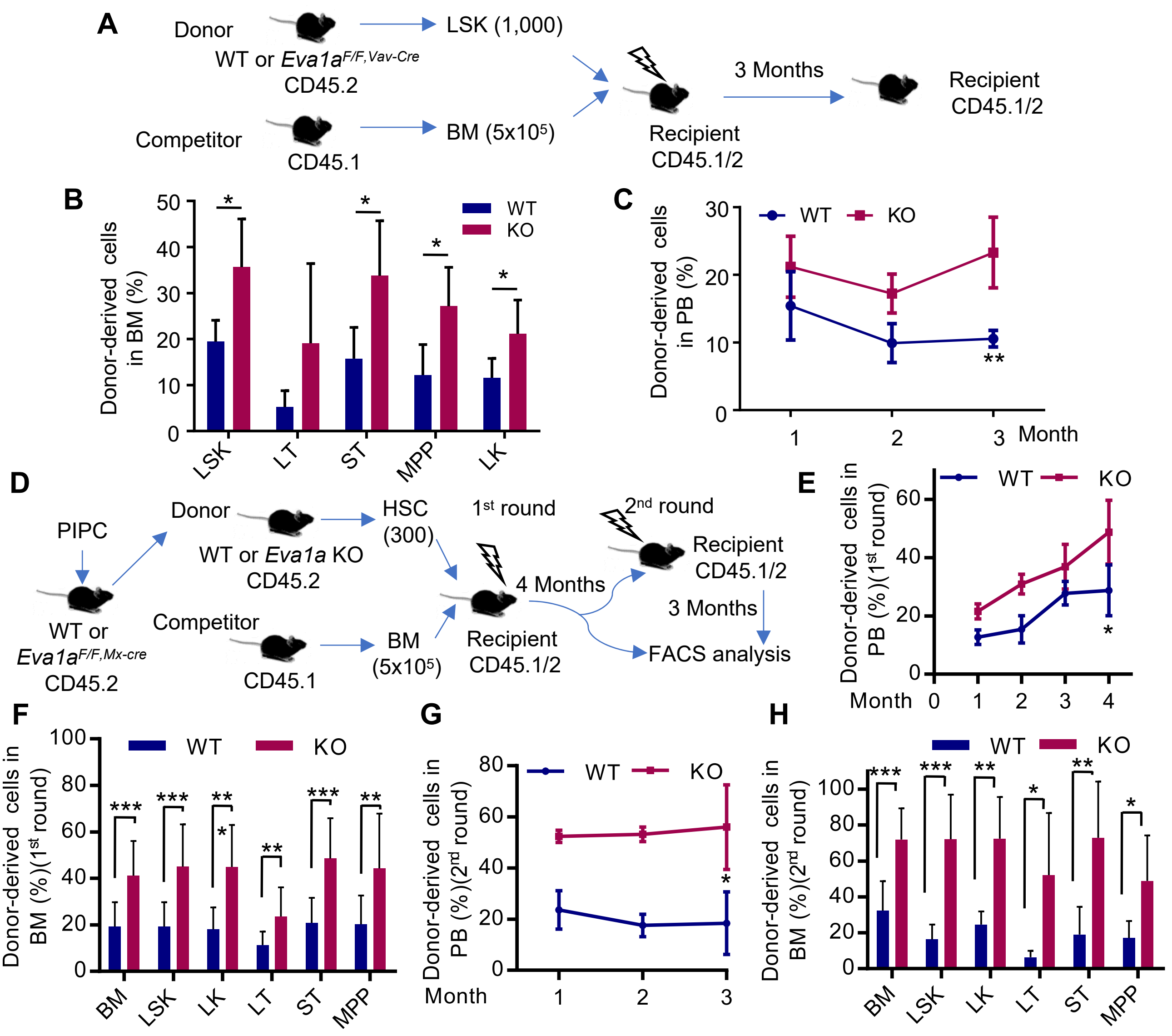

Figure S3. *Eva1a* deletion enhances the repopulation capacity of HSPCs. (A) Experimental schematic for competitive transplantation with WT and *Eva1a*$^{F/F,Vav\text{-}Cre}$ LSK cells (results in B-C). (B) The percentage of donor-derived LSK(Lin$^-$cKit$^+$Sca1$^+$), LT(Flt3$^-$CD34$^-$LSK), ST(Flt3$^-$CD34$^+$LSK), MPP(Flt3$^+$CD34$^+$LSK) and LK(Lin$^-$cKit$^+$Sca1$^-$) cells in the recipients at 3rd month (n=4~5). (C) The percentage of donor-derived cells in PB after transplantation (n=4~6). (D) Experimental schematic for serial competitive transplantation with SLAM-HSC cells from WT and Eva1a$^{F/F,Mx\text{-}Cre}$ mice (results in E-H). (E, G) The percentage of donor-derived cells in PB at the indicated time points during 1st round (E) and 2nd round (G) of the transplantation (n=3~5). (F, H) The percentage of donor-derived BM, LSK, LK, LT, ST and MPP cells in the primary recipients after transplantation 4 months (F) and the secondary recipients (H) after transplantation 3 months (n=7~12). Data are presented as mean ± SD, (*p < 0.05, **p < 0.01, and ***p < 0.001).

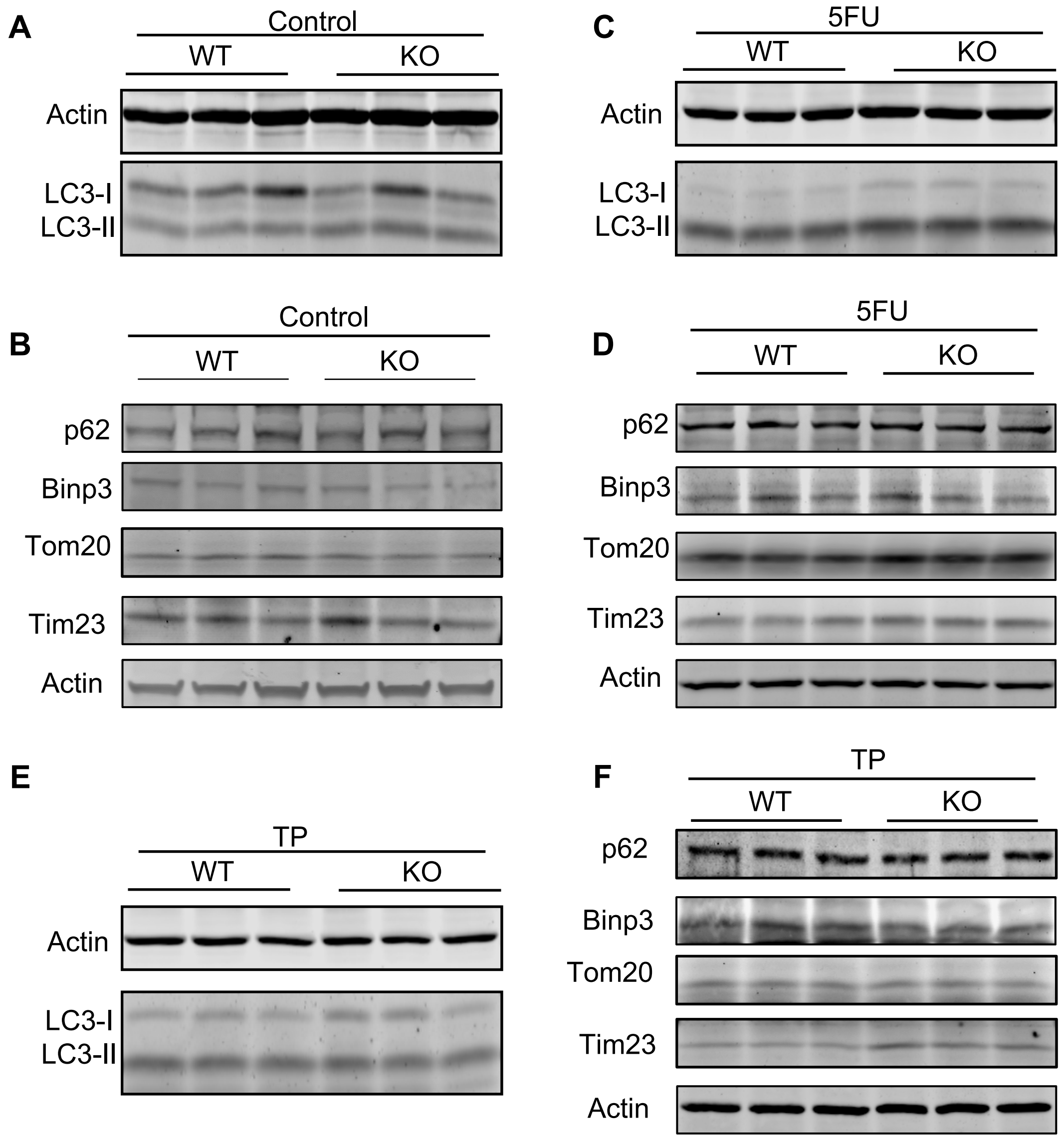

Figure S4. *Eva1a* deficiency does not affect the autophagosome formation of HSPCs. (A, B) Western blot analysis of the cellular LC3 (A), p62, Binp3, Tom20 and Tim23 (B) protein levels in Lin^- ($Lineage^-$) cells from WT and $Eva1a^{F/F, Vav\text{-}Cre}$ mice. (C, D) WT and $Eva1a^{F/F, Vav\text{-}Cre}$ mice were treated with a single dose of 150mg/kg 5FU, and the LC3 (C), p62, Binp3, Tom20 and Tim23 (D) protein levels in Lin^- cells were assessed by Western blot at day 8 after the 5FU treatment. (E, F) CD45.1 mice after lethal irradiation were transplanted with 2×10^6 BM cells from WT and $Eva1a^{F/F, Vav\text{-}Cre}$ mice for 14 days, then the cellular LC3 (E), p62, Binp3, Tom20 and Tim23 (F) protein levels of the donor-derived Lin^- cells were assessed by Western blot.

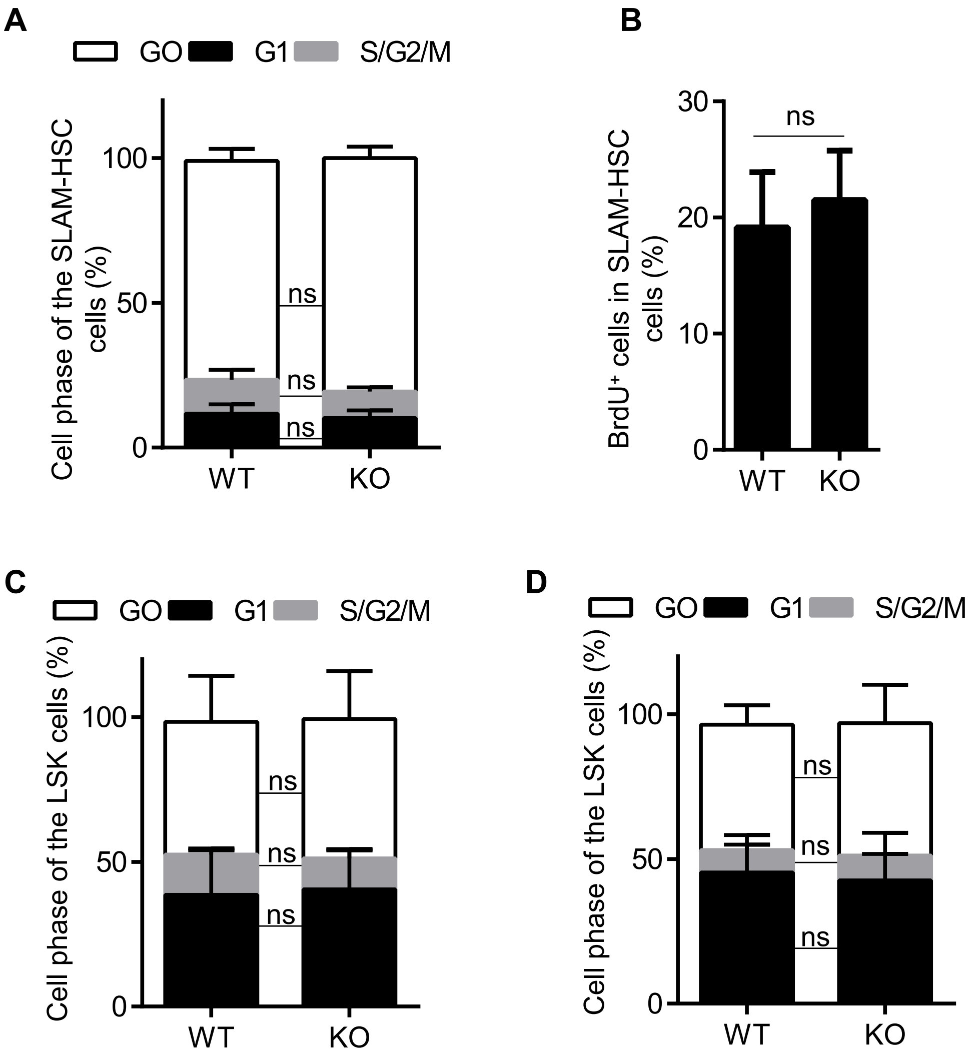

Figure S5. ***Eva1*a deficiency does not affect HSPC proliferation**. (A) The percentage of G0/ G1/ S/G2/M phase in SLAM-HSC cells from WT and *Eva1a*$^{F/F, Vav\text{-}Cre}$ mice were assessed by Ki67 staining. (B) The percentage of BrdU-positive cells in SLAM-HSC cells from WT and *Eva1a*$^{F/F, Vav\text{-}Cre}$ mice were assessed by BrdU labeling. (C) The percentage of G0/ G1/ S/G2/M phase were assessed by Ki67 staining in LSK cells from WT and *Eva1a*$^{F/F, Vav\text{-}Cre}$ mice after 5FU treatment 8 days. (D) CD45.1 mice after lethal irradiation were transplanted with 1X 10^6 BM cells from WT and *Eva1a*$^{F/F, Vav\text{-}Cre}$ mice, then the percentage of G0/ G1/ S/G2/M phase were assessed by Ki67 staining in donor-derived LSK cells after transplantation 14 days. Data are presented as mean ± SD, (n=3~5), p values less than 0.05 was considered to statistical significance.

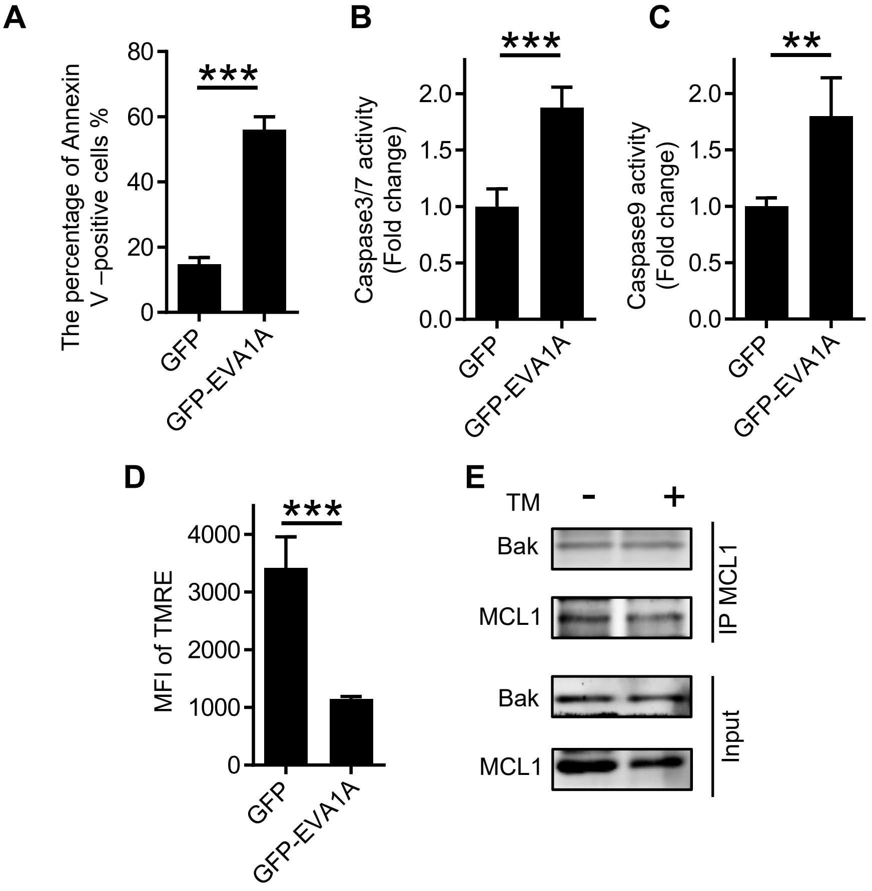

Figure S6. EVA1A overexpression stimulates apoptosis. (A) The percentage of Annexin V positive cell in cells expressing GFP or GFP-EVA1A, (n=5). (B) Caspase 3/7 activity of the cells expressing GFP or GFP-EVA1A, (n=5). (C) Caspase 9 activity of the cells expressing GFP or GFP-EVA1A, (n=5). (D) The median fluorescence intensity (MFI) of TMRE in cells expressing GFP or GFP-EVA1A, (n=5). (E) *Eva1a* knockout MEF cells were treated with Tunicamycin (TM) and immunoprecipitated with anti-MCL1 antibody, then the immunocomplexes were analyzed by Western blot. All the statistical data are presented as mean ± SD, (**$p < 0.01$, and ***$p < 0.001$).

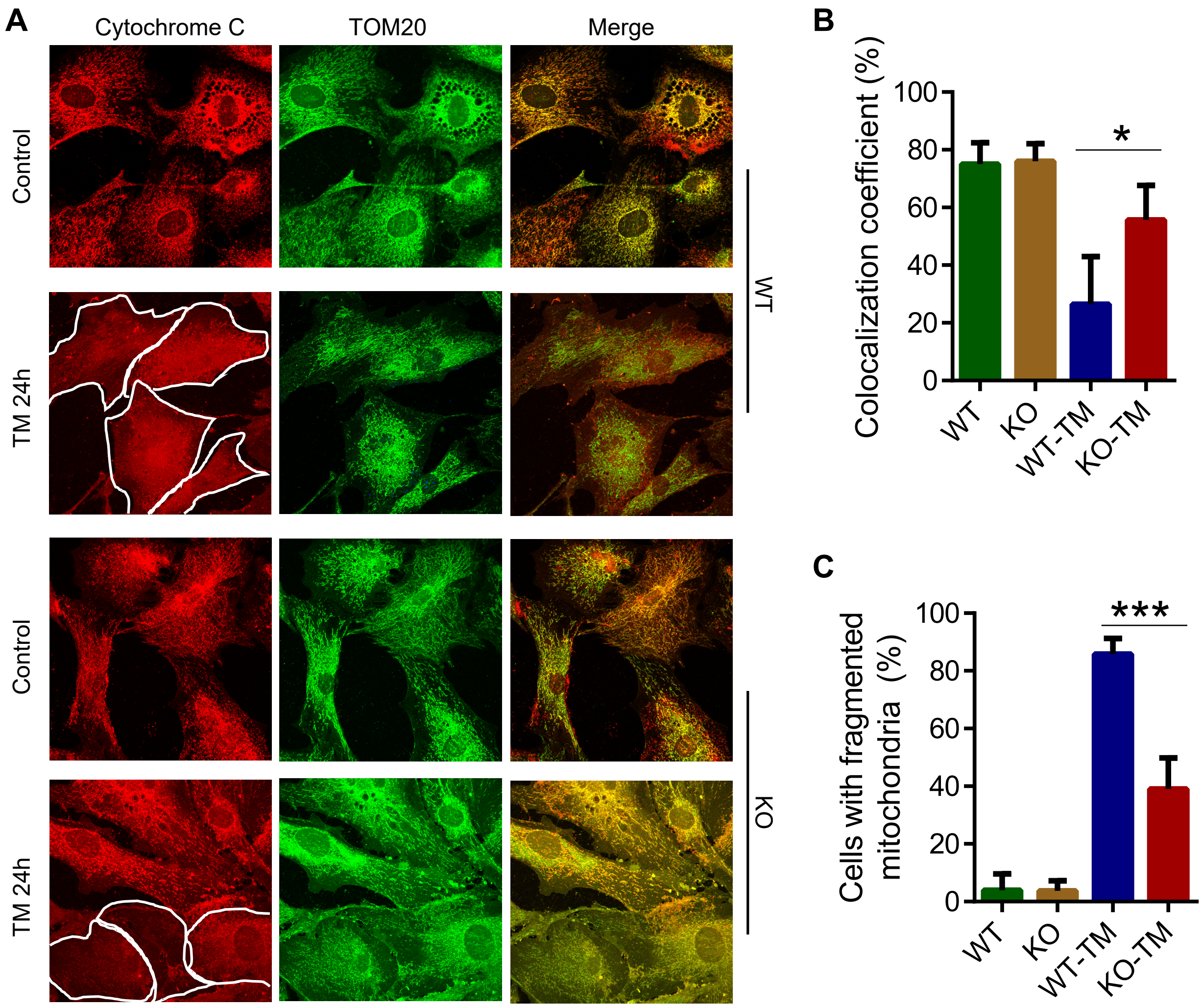

Figure S7. *Eva1a* deficient inhibited Tunicamycin induced mitochondria-associated apoptosis. (A, B) Representative image (A) and quantification (B) showing the colocalization of Cytochrome C and TOM20 in WT and *Eva1a* knockout MEF cells treated without or with 3 μg/ml Tunicamycin (TM) for 24h. The colocalization coefficient was represented as percentage of signals of Cytochrome C that were positive for TOM20. Quantifications were performed using Volocity software (n>15cells). (C) WT and *Eva1a* knockout MEF cells treated as in (A), and the cells with fragmented mitochondria were assessed (n>50cells). All the statistical data are presented as mean ± SD, (*$p < 0.05$, and ***$p < 0.001$).

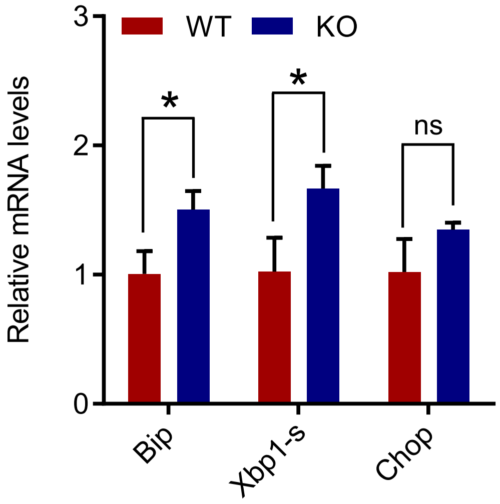

Figure S8. ***Eva1a*** **deficiency results in a mild expression increase of the UPR target genes.**

The relative mRNA level of the UPR target genes in LSK cells from WT and *Eva1a*$^{F/F,Vav\text{-}Cre}$ mice. Data are presented as mean ± SD, (n=3), (*$p < 0.05$).

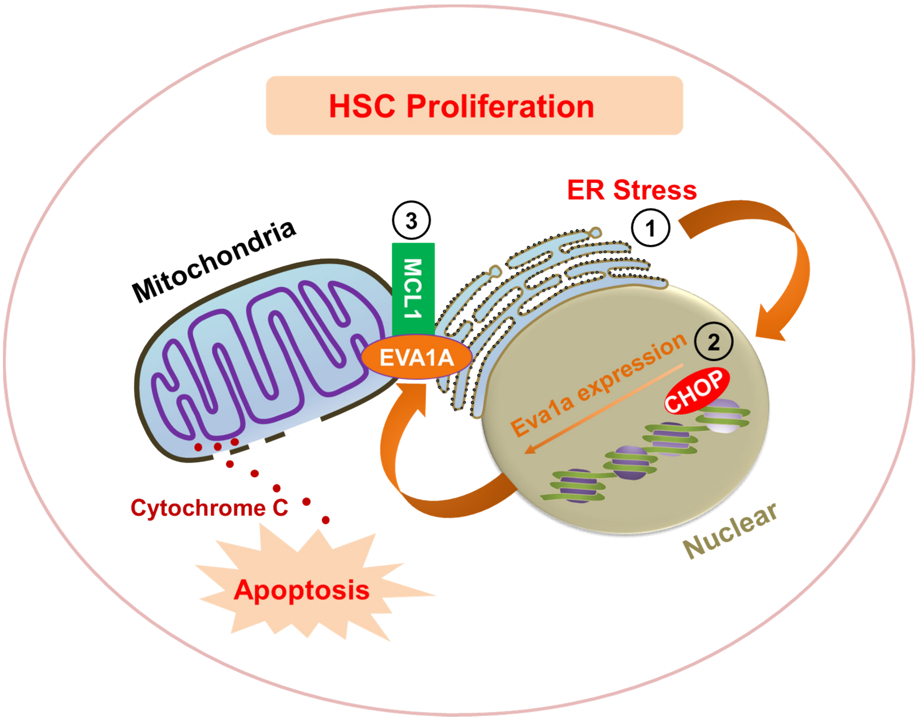

Figure S9. A model for EVA1A-regulated ER stress-induced apoptosis under hematopoietic regeneration. 1) Hematopoietic regeneration induces ER stress, which limits the repopulation capacity of the activated HSPCs by upregulating EVA1A expression. 2) In response to ER stress, CHOP upregulates EVA1A expression exerting ER stress-induced apoptosis. 3) EVA1A triggers ER-Mitochondria mediated apoptosis by interacting with MCL1.
