# Supplementary figures and images for: EVA1A regulates hematopoietic stem cell regeneration via ER-mitochondria mediated apoptosis
Source: Cell Death Dis. 2023 Jan 30;14(1):71. doi: 10.1038/s41419-023-05559-9 (PMC9887066; doi:10.1038/s41419-023-05559-9)

**Fig1-A**

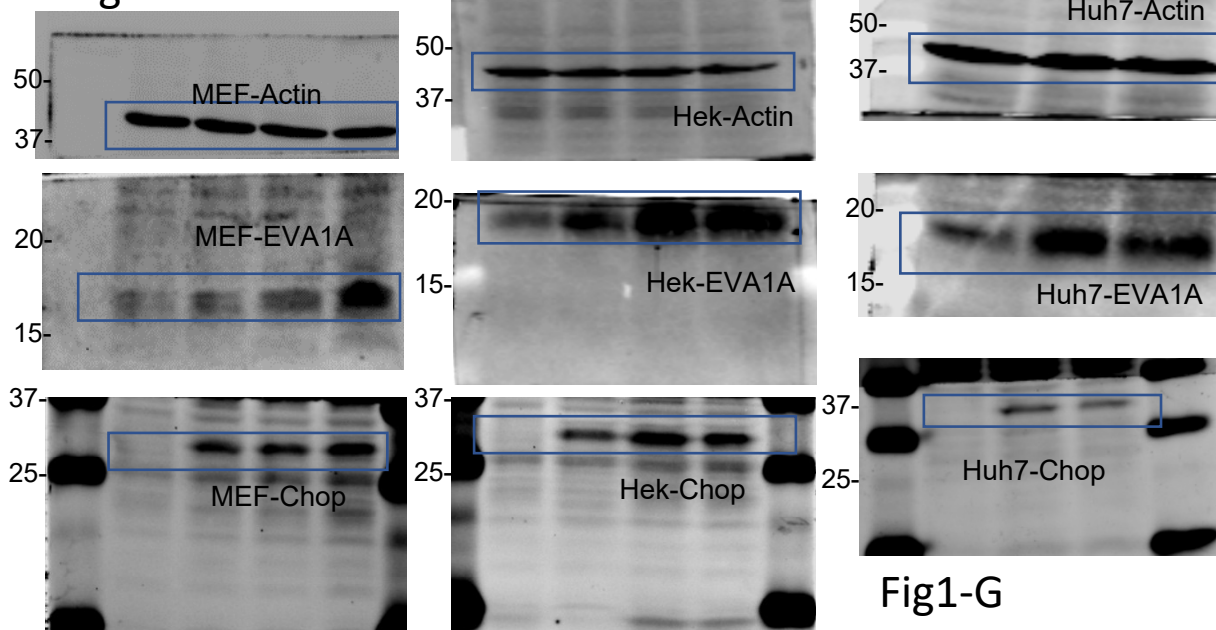

**Fig1-B**

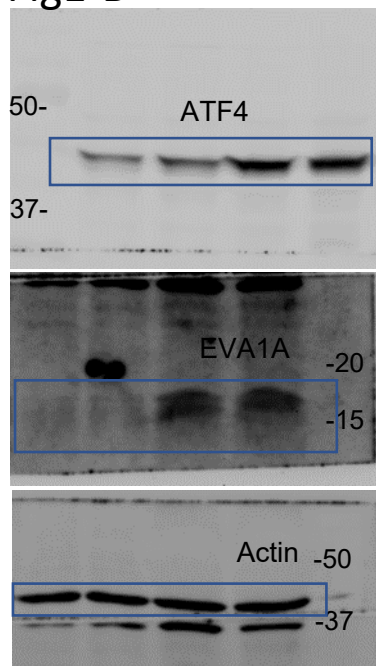

**Fig1-E**

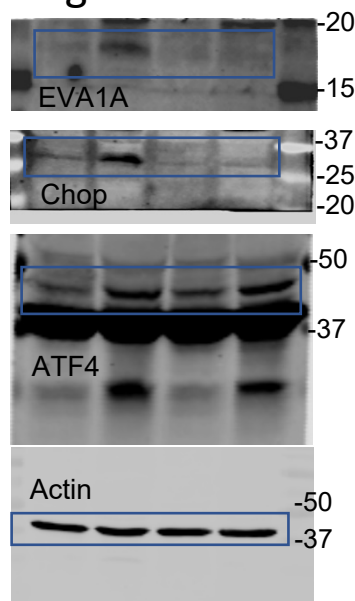

**Fig1-F**

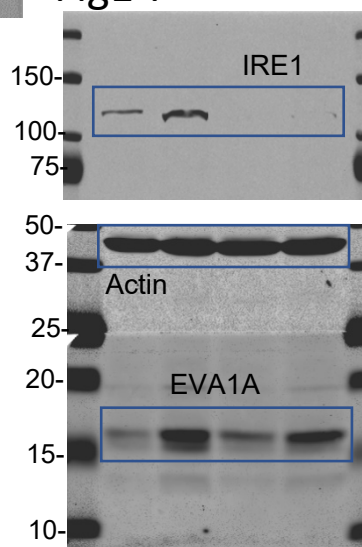

**Fig1-G**

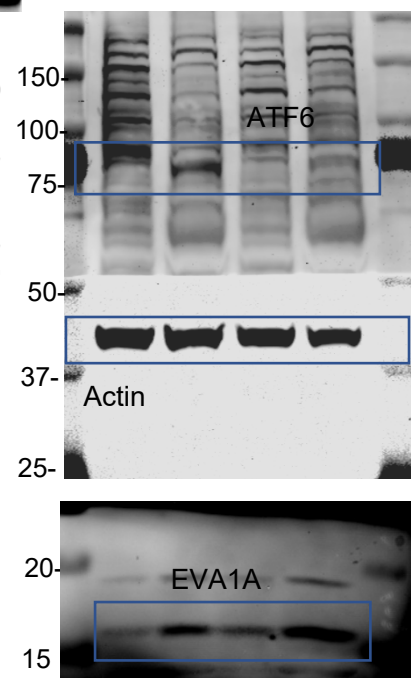

**Fig1-H**

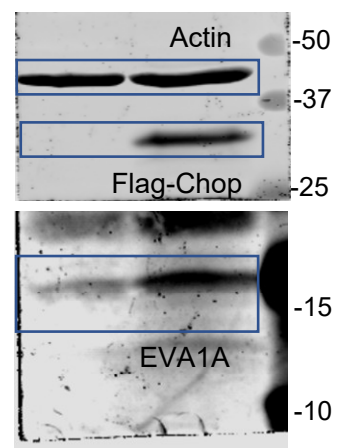

Fig2-D

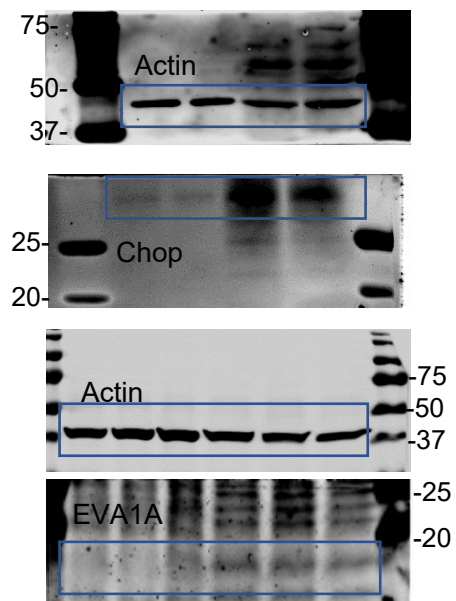

Fig2-F

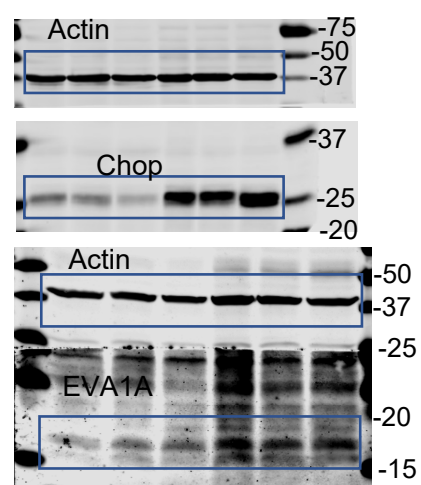

Fig6-B

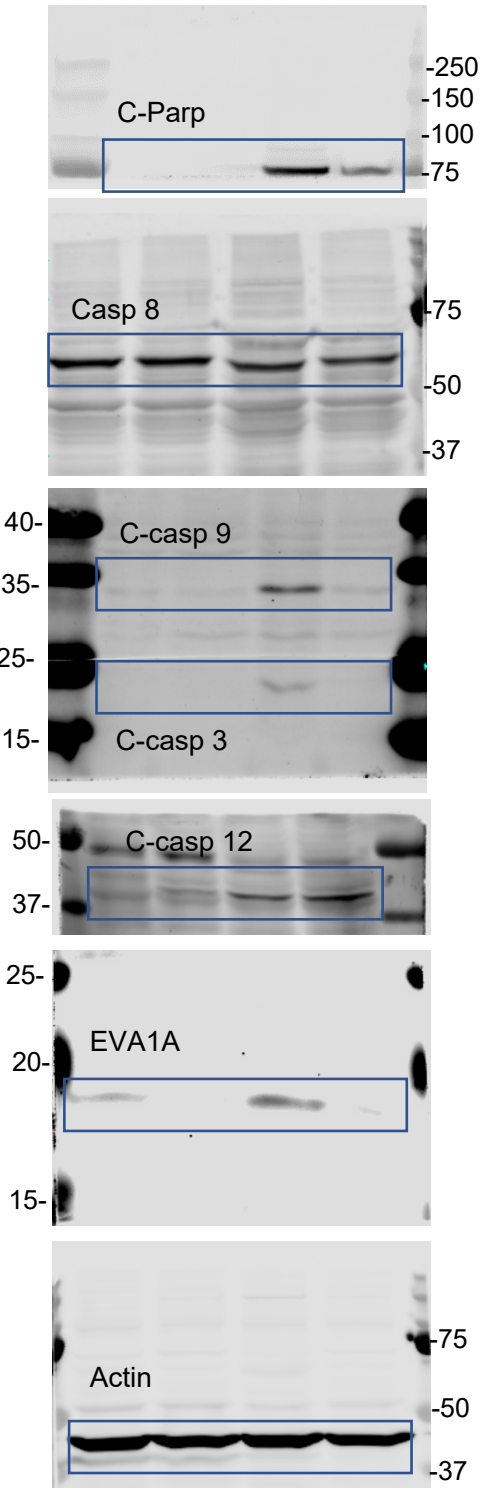

Fig6-D

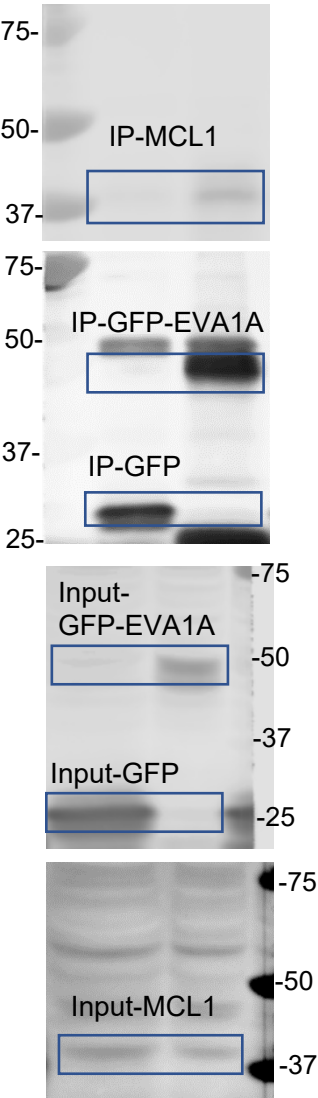

Fig6-E

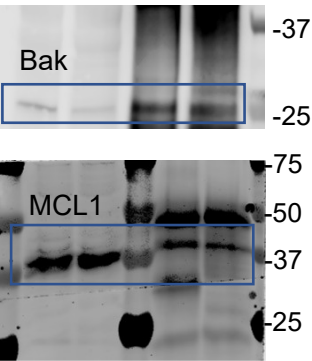

FigS4-A

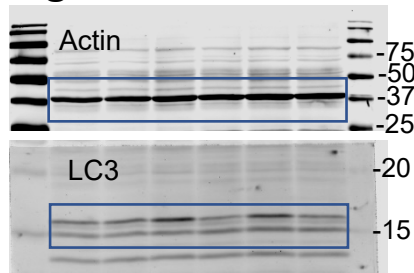

FigS4-B

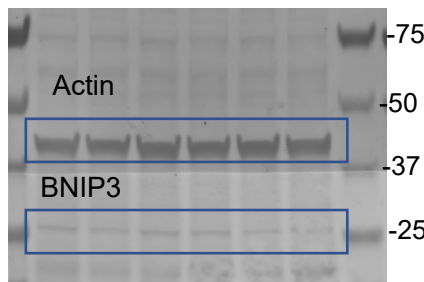

FigS4-D

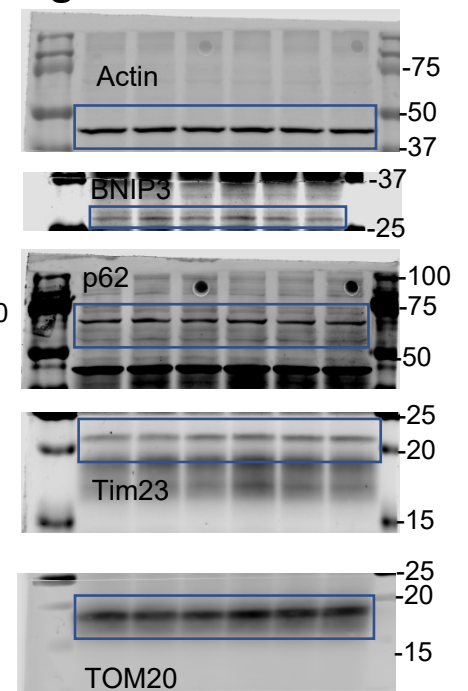

FigS4-C

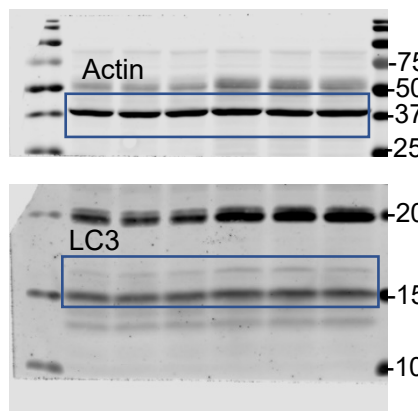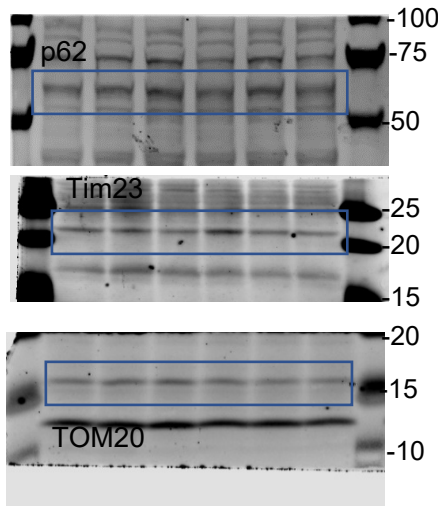

FigS4-E

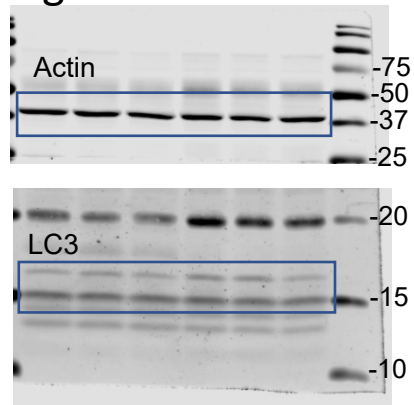

FigS4-F

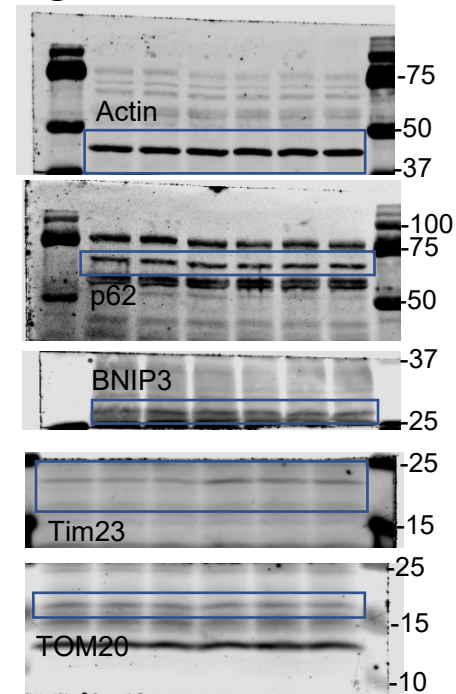

FigS6-E

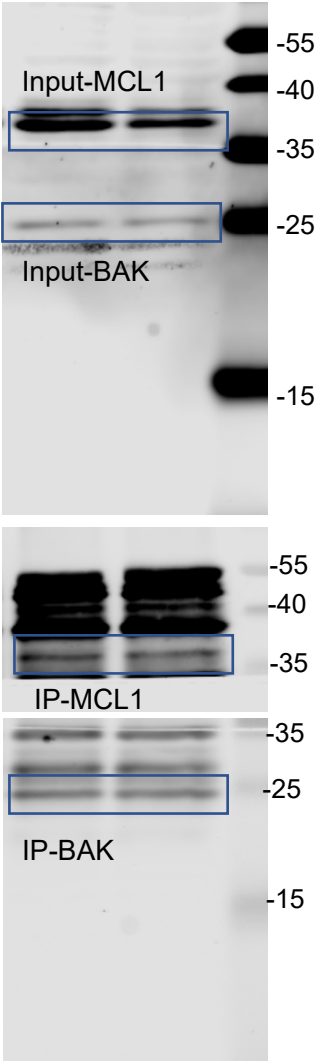

Supplement: Supplementary file 2 — Original Data File [file 41419_2023_5559_MOESM2_ESM.pdf]
